# Adjuvant treatment with yupingfeng granules for recurrent respiratory tract infections in children: A systematic review and meta-analysis

**DOI:** 10.3389/fped.2022.1005745

**Published:** 2022-12-21

**Authors:** Lu Zhang, Xuqiong Wang, Dong Wang, Yinling Guo, Xinying Zhou, Haiyan Yu

**Affiliations:** School of Basic Medical Sciences, Chengdu University of Traditional Chinese Medicine, Chengdu, China

**Keywords:** yupingfeng granules, recurrent respiratory tract infections, children, systematic review, meta-analysis

## Abstract

**Background:**

Yupingfeng granules (YPFG) contribute to various chronic respiratory infections. Several clinical studies have evaluated its efficacy and safety in treating recurrent respiratory tract infections (RRTIs) in children. However, the evidence for its use has not been conclusively proven.

**Objective:**

The aim of this study was to demonstrate the efficacy and safety of YPFG in the adjuvant treatment of RRTIs in children.

**Methods:**

We searched PubMed, Embase, Web of Science, Cochrane Library, Clinical Trials, Chinese Clinical Trial Registry, Sinomad, China National Knowledge Infrastructure (CNKI), Wanfang Database, and Chinese Scientific Journals Database (VIP) for randomized controlled trials (RCTs) of YPFG adjuvant therapy for children with RRTIs as of September 1, 2022. We screened the literature for inclusion and exclusion criteria, assessed the quality of each included literature, and then extracted data from each study for this systematic review and meta-analysis.

**Results:**

A total of 17 RCTs were included. Data analysis showed that the total clinical response rate in the YPFG group was significantly higher than that in the control group [risk ratio (RR) = 1.18, 95%CI (1.12, 1.24), *I*^2 ^= 39%, *P* < 0.00001]. Compared with the control group, three serum immunoglobulin levels were significantly increased in the YPFG group: IgA level [standardized mean difference (SMD) = 1.23, 95%CI (0.68, 1.78), *I*^2^ = 95%, *P* *< 0.0001*]; IgM level [SMD = 0.85, 95%CI (0.35, 1.35), *I*^2^ = 93%, *P* = 0.0009]; IgG level [SMD = 1.06, 95%CI (0.65, 1.47), *I*^2^ = 91%, *P* < 0.00001]. The TNF-α level was significantly lower in the YPFG group [SMD = −1.03, 95%CI (−1.55, −0.51), *I*^2^ = 84%, *P *= 0.0001] compared with the control group.

**Conclusions:**

In summary, adjuvant YPFG therapy improves clinical efficacy and immunity in children with RRTIs. However, the effectiveness and safety of YPFG remain to be further verified.

**Systematic review registration:**

[https://inplasy.com/inplasy-2022-3-0150/], identifier [INPLASY202230150].

## Introduction

RRTIs are among the most common diseases in pediatrics and include upper or lower respiratory tract infections (RTIs), which occur frequently every year (1). However, there is no global consensus on the exact definition of disease recurrence (2). Currently, pediatric RRTIs are generally aimed at ≥8 respiratory infections per year in preschool children (<3 years of age) without underlying medical conditions and ≥6 respiratory infections per year in children over 3 years of age (3). In China, children can be diagnosed with RRTIs when the number of episodes in a year exceeds the standard frequency (4).

RRTIs in children have a high incidence and long duration (3), are the leading risk factor for death and disability in preschool children (5), severely affect their physical and mental health, and pose a significant medical burden on families and society (6). It is estimated that approximately 25%–45% of children require surgery for severe RRTIs (7), and a prompt diagnosis of the disease is essential to initiate appropriate treatment and minimize irreversible changes.

Viral and bacterial infections are major pathogenic factors (8, 9). An immature immune system, allergies, and air pollution can also play a role (6). Since various immune disorders are common in RRTIs, and immunodeficiency may be an important predisposing factor (10). Children with RRTIs may have increased myelomonocytic suppressor cells (MDSCs) and reduced CD8^+^ T-cell function (11). About 65% of cases suffer from humoral immune disorders, usually characterized by abnormal IgG, IgA, and IgM status, leading to infection and respiratory dysfunction (12). Recurrent episodes and severe respiratory infections are the most common clinical manifestation and diagnostic basis of childhood primary immunodeficiency disease (PIDD). Aggressive prophylaxis, antibiotics, and immunoglobulin replacement are critical to improving outcomes (13).

Clinically effective drugs for pediatric RRTIs are still unavailable, vaccines are difficult to prevent multiple viral infections, and antibiotic resistance has become a common side effect of drugs (14, 15). Pidotimod is safe as an immunomodulator, but it has limited utility and is expensive to treat (2). YPFG is a proprietary Chinese medicine isolated from Huang Qi, Fang Feng, and Bai Zhu. It is derived from the famous Yupingfeng powder with hundreds of years of clinical verification history. YPFG multi-targeted, multi-pathway immunomodulatory effects and has a wide range of therapeutic products to treat various respiratory infections and immune diseases (16–18). YPFG has good efficacy (19) and clear cost-effectiveness benefits in treating pediatric RRTIs (20).

Potential adjuvant therapy plays a beneficial role in preventing and managing pediatric RRTIs (21). Conclusions regarding YPFG treatment are not entirely consistent (22, 23), so we conducted a meta-analysis to verify the efficacy of YPFG and explore possible reasons for the differences between studies.

## Methods and registration

We performed this study (Supplementary File S1) according to the Preferred Reporting Project for Systematic Reviews and Meta-Analysis (PRISMA) (24), whose protocol is registered on the INPLASY platform (INPLASY202230150). The study was based on a systematic review of the published literature. It does not affect patient privacy or the right to information and therefore does not require ethics committee review and approval.

### Literature search

Two reviewers (XQW and DW) searched 10 electronic databases: PubMed, Embase, Web of Science, Cochrane Library, Clinical Trials, Chinese Clinical Trial Registry, Sinomad, China National Knowledge Infrastructure (CNKI), Wanfang Database, and Chinese Scientific Journals Database (VIP). Each database was searched from the establishment of the database to September 1, 2022, only in Chinese and English. Two reviewers used a combined index term of subject and free words. References included in the study and related analysis were also manually searched to determine the completeness of the search. We searched the literature using the following keywords: (yupingfeng or Yu Ping Feng San or YPFS herbal formulation or Yupingfeng Powder granules or Yupingfeng Powder or Yupingfeng granules or YuPingFeng granules or Yupingfeng Granule or yupingfeng formula or Jade-Screen powder or Yu Ping) and (infant or child or pediatrics or Infants or children) and (Respiratory Tract Infections or Respiratory Infections or recurrent respiratory tract infections).

### Study selection

Based on inclusion and exclusion criteria, two researchers (XYZ and HYY) independently screened all searched literature using Endnote software. They exchanged checks to confirm the accuracy and consulted, a third researcher (YLG) to resolve differences.

### Inclusion and exclusion criteria

(1) Population: Children with RRTIs of any age (especially preschool children were eligible), but children with co-morbidities or congenital immunodeficiency must be excluded.
(2) Intervention: The experimental group received YPFG adjuvant therapy according to the regulations of each study, with complete drug information and accurate dose application. Both groups of children received conventional treatment in the acute stage of respiratory tract infection, including antipyretic, antitussive, and antiviral therapy. Both the experimental group and the control group, combined with other traditional Chinese medicine (TCM) treatments such as acupuncture and moxibustion, should be excluded.
(3) Comparison: The control group received a placebo or other drugs, except TCM.
(4) Outcomes: Total clinical effective rate, serum immunoglobulin levels of IgA, IgG or IgM, TNF-α level, and adverse events.
(5) Study design: Clinical RCTs only. Other literary styles such as animal experiments, case reports, reviews, systematic reviews, communications, and retrospective studies, must be excluded. At the same time, unpublished research and conference papers were also excluded.

### Data extraction

Two researchers (XYZ and HYY) independently reviewed titles, abstracts, and full-text contents of the literature and consulted the third researcher when necessary (YLG). The following data were extracted independently: study characteristics (name of the first author, year of publication, and type of study); baseline characteristics (sample size, age, and disease duration); therapeutic regimen (interventions and time of both groups); outcomes (total clinical effective rate, serum immunoglobulin levels of IgA, IgG or IgM, TNF-α level, and adverse events).

### Quality assessment

Two reviewers (XQW and LZ) independently assessed the methodological quality of included studies using Review Manager 5.3 according to the Cochrane Risk of Bias tool (25). Each study was assessed using seven biased projects: (1) randomization methods; (2) attribution obfuscation; (3) blinding of designers and participants; (4) blinded assessment results; (5) completeness of data; (6) publication bias; and (7) other bias. Each bias contained three levels of risk: high risk, unclear, or low risk. Any disputes were resolved in consultation with the researcher (YLG).

### Statistical analysis

Meta-analyses for each outcome measure were done independently using Review Manager 5.3 and Stata 12.0. A risk ratio (RR) with a 95% confidence interval (CI) was used for dichotomous data. We obtained continuous results using weighted mean differences (WMD) and standardized mean differences (SMD) to remove the effect of absolute values between studies. SMD eliminated the effect of different measurement units between studies. The difference in outcome after the trial intervention was calculated as the difference between the baseline and endpoint values. Since the included studies did not report the baseline-final correlation coefficient (Corr), we chose the commonly utilized value of 0.5 as the Corr value (26, 27). We performed a meta-analysis when at least four studies included the same outcome measure and showed 95% CIs for statistical results, with *P* < 0.05 representing statistical significance.

Data heterogeneity was assessed using the index of inconsistency (*I*^2^), with moderate heterogeneity reported when *I*^2^ > 50% and high heterogeneity when *I*^2^ > 75% (28). Considering sampling error and sample heterogeneity, it was necessary to try to balance the actual effect sizes of each study, so we used a random effects model in this meta-analysis (29). To analyze the source of heterogeneity, a subgroup analysis was performed based on control groups, test groups, and treatment duration of YPFG. Egger test and visual symmetry of funnel plots were used to assess the possibility of publication bias. Sensitivity analysis assesses statistical stability by excluding included studies on a case-by-case basis. The evidence certainty of all RCTs was assessed using the GRADE classification method (30). Corresponding quality-of-evidence recommendations are then provided.

## Results

### Literature search

**Figure 1:**
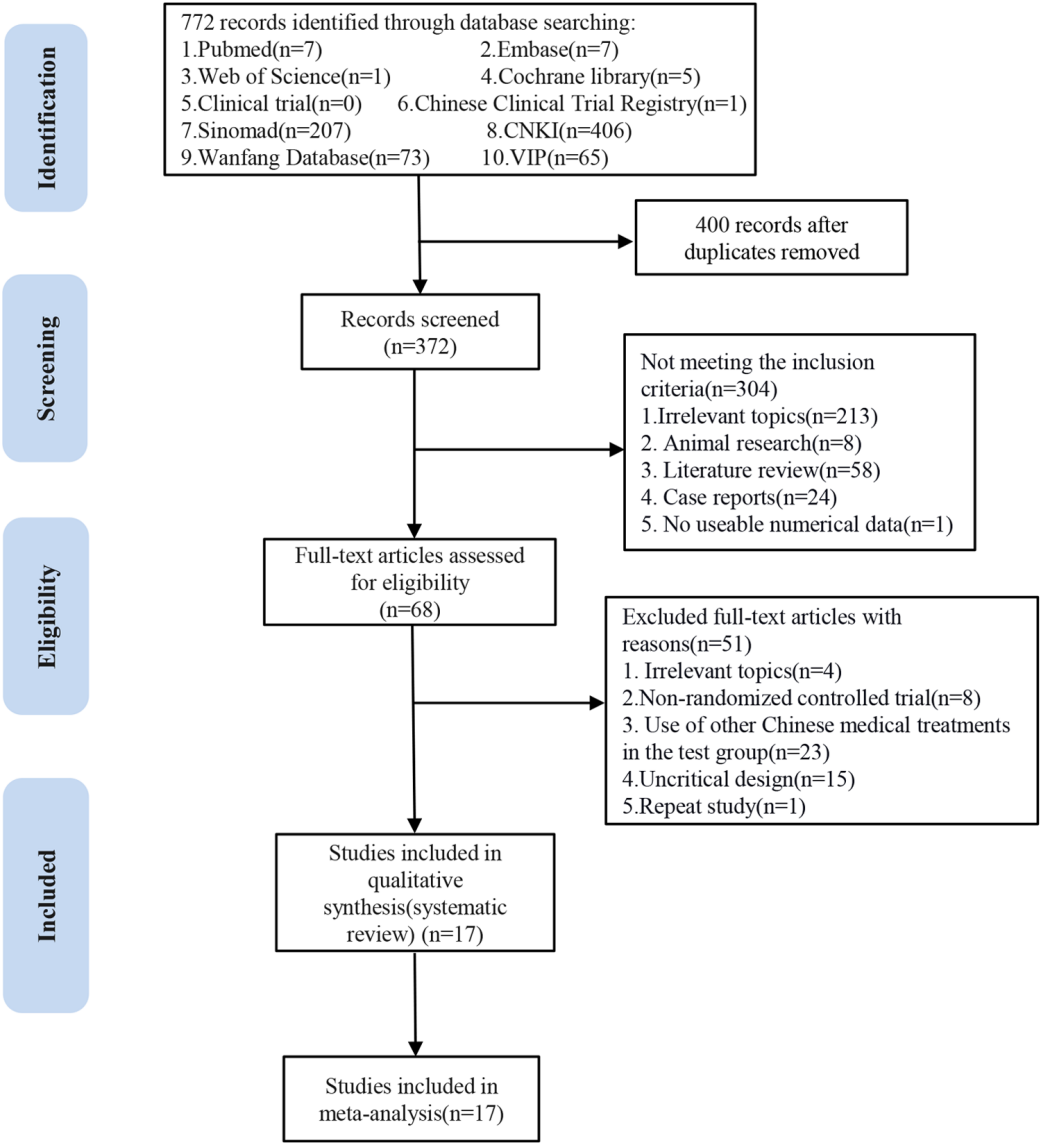
Flow chart of the study screening process.

A total of 772 published papers from clinical trials were retrieved, including 406 identified by CNKI, 73 identified by WanFang, 207 identified by Sinomad, 65 identified by VIP in the Chinese database, 7 identified by PubMed, 7 identified by Embase, 1 identified by Web of Science, 5 identified by Cochrane Library, and 1 identified by Chinese Clinical Trial Registry in English database. Four hundred duplicate search results were removed. First, 372 articles were initially screened by browsing the titles and abstracts; then, the remaining 68 articles were scanned in total. Finally, 17 articles were identified for inclusion in our study. All RCTs were completed in China, and the results were published in Chinese. Figure 1 illustrates the literature screening process.

### Quality assessment

**Figure 2:**
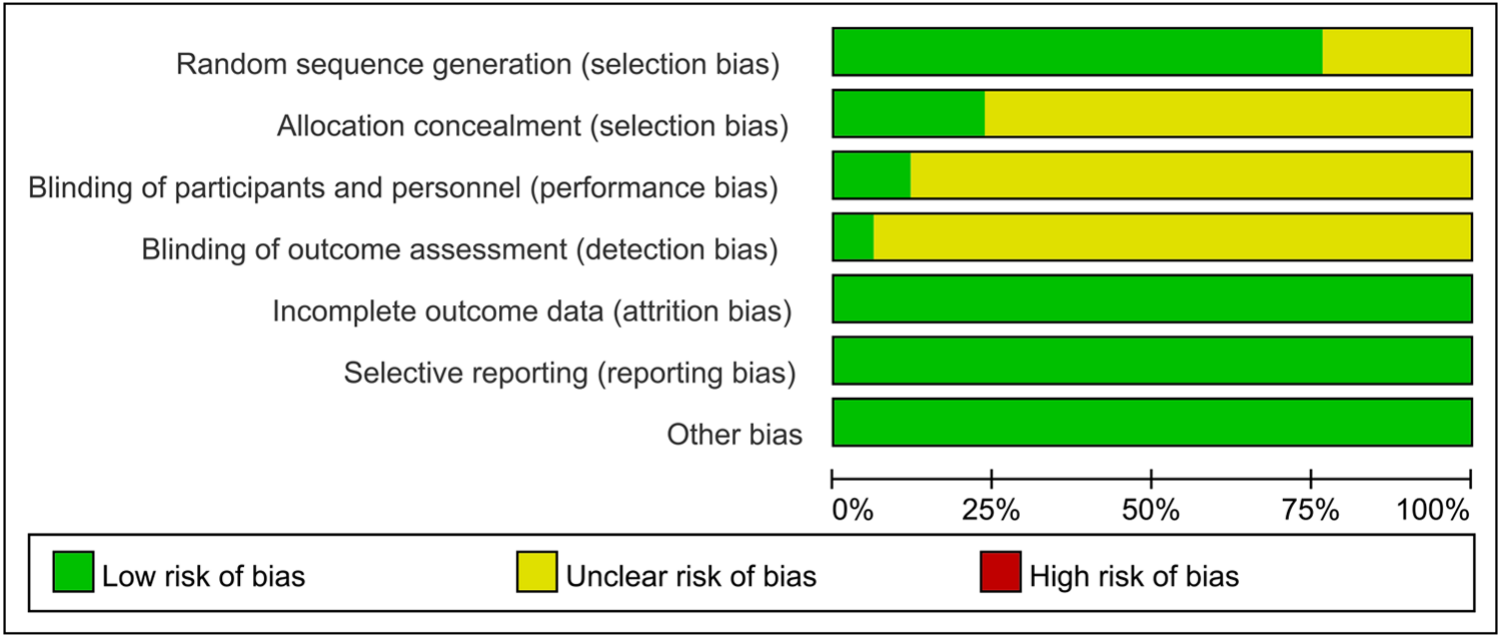
The plot of risk bias ratio for included studies.

**Figure 3:**
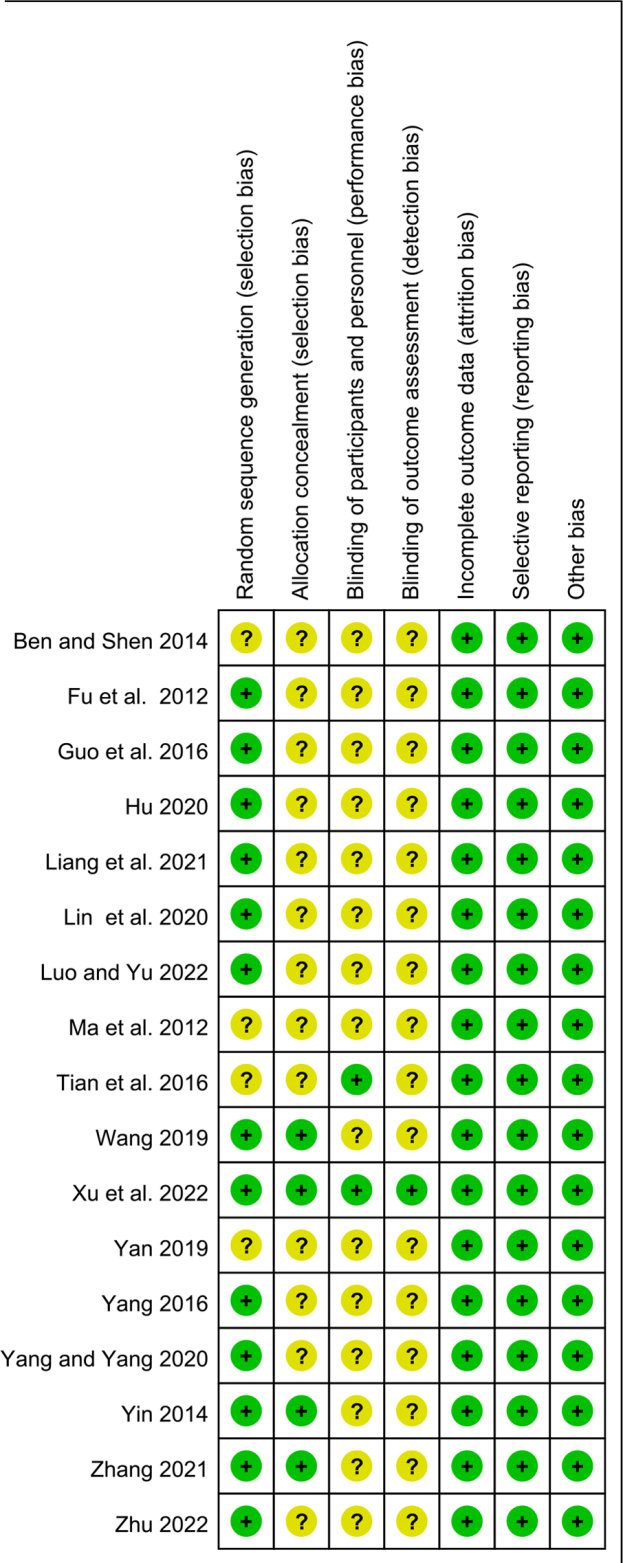
The summary of risk of bias for included studies.

The risk of bias assessment for the included studies is shown in Figures 2, 3. Most studies generated random sequences through random number tables or illustrate them using randomization. The study by Yin reported the allocation concealment method (31). Two studies reported blinded schemes to generate random sequences: Zhang's study used a double-blind process (32), and Tian et al. used a single-blind method (33). Xu et al.'s study (23) was the only multicenter, double-blind, double-simulation RCT. Outcome indicators for each trial were fully described in the literature. The study by Fu et al. reported 4 withdrawals in the experimental group due to medication noncompliance and 5 withdrawals in the control group due to irregular follow up (34). Except for the study by Xu et al. (23), all RCTs were published in Chinese, so there is a potential risk of regional bias. In conclusion, most studies are classified as unclear in most studies due to insufficient information on study design and conduct.

### Study characteristics

Finally, we included 17 trials (22, 23, 31–45) for systematic review and meta-analysis. Participants were children aged 1 to 9 years with a diagnosis of RRTIs. A total of 1,970 children were enrolled, including 1,084 boys and 886 girls. The duration of recurrent respiratory infections in children varies widely because some studies recorded the history, that is, the time from initial onset to recent start. In contrast, others only recorded the most recent course of illness before admission. The number of recurrences of RTIs ranged from 3 to 13 per year. The duration of treatment with YPFG in the experiment group ranged from 7 days to 4 months. Regarding the interventions in the control group, eight studies (22, 23, 33, 35, 40–42, 44) used immunomodulators and the other nine used only conventional treatment. The main characteristics and intervention protocols of each study are detailed in Table 1.

**Table 1:** Characteristics of the included literatures.

Study	Type	Participant characteristics			Test	Control	Treatment duration	Outcomes	Adverse events	Follow-up
		Sample size, sex (boy/girl), Age (T/C), year	Disease duration	Recurrence times of infection						
Yin 2014	RCT	120 (63/57) T:31.37 ± 7.817 C:30.05 ± 8.096 (months)	T:3.03 ± 1.339 C:3.03 ± 1.615 (Pre-hospital course, days)	T:5.196 ± 1.271 C:4.966 ± 1.160 (times/ half a year)	1.YPFG (children ages 1–3, 2.5 g, TID; > 3 years old, 5 g, TID) 2. Routine treatment	1. Routine treatment	1 month	1. Total effective rate 2. STCMS 3. Signs and Symptoms Score 4. Serum immunoglobulin levels before and after treatment (IgA, IgG, IgM) 5. Number of infections within 6 months	No significant adverse events	6 months
Ben and Shen 2014	RCT	98 (52/46) T:3.8 ± 2.6 C:4.5 ± 2.3	NR	NR	1. YPFG (Children ages < 3, 2.5 g, TID;≥3 years old, 5 g, TID) 2. Control	1. Spleen-aminopeptide (2 mg/day, QOD) 2. Routine treatment	2 months	1. Total effective rate 2. Serum immunoglobulin levels before and after treatment (IgA, IgG, IgM)	NR	1 year
Yang 2016	RCT	110 (60/50) T:2.9 ± 1.5 C:3.1 ± 1.7	NR	NR	1. YPFG (Children ages 1–3, 2.5 g, TID;≥3 years old, 5 g, TID) 2. Routine treatment	1. Routine treatment	1 month	1. Total effective rate 2. Post-treatment serum immunoglobulin levels (IgA, IgG, IgM)	No significant adverse events	NR
Luo and Yu 2022	RCT	60 (31/29) T:3. 80 ± 1. 55 C:3. 51 ± 1. 74	T:18. 64 ± 2. 59 C:17. 34 ± 3. 49 (months)	T:8. 06 ± 1. 69 C:8. 01 ± 2. 54 (times/year)	1. YPFG (5 g/packet,5 g,TID) 2. Control	1.Routine treatment 2.Pidotimod oral solution (7 ml,take one dose in the morning and one in the evening)	2 weeks	1. Total effective rate 2. Serum immunoglobulin levels before and after treatment (IgA, IgG, IgM) 3. T-cell subpopulation index levels before and after treatment (CD3^+^,CD4^+^,CD8^+^) 4. Duration of clinical symptoms (Cough, tonsillar erythema, fever and wet rales) 5. Incidence of adverse reactions	T:2 (6.67%): 1 nausea, 1 vomiting C:8 (26. 67%): 3 cases of nausea, 5 vomiting	6 months
Yang and Yang 2020	RCT	158 (82/76) T:3.7 ± 1.2 C:3.5 ± 1.1	T:14.5 ± 3.3 C:14.2 ± 3.1 (months)	NR	1. YPFG (Children < 10 kg, 1. 5 g, TID; 10–20 kg, 2. 5 g, BID; 20–30 kg, 2.5 g, TID; ≥30 kg children, 5 g, TID) 2. Calcium and zinc gluconates oral solution 3. Routine treatment	1. Pidotimod granule (20 ml, BID; after 2 weeks, 20 ml, QD) 2. Calcium and zinc gluconates oral solution (5–10 ml, BID or TID) 3. Routine treatment	2 months	1. Serum immunoglobulin levels before and after treatment (IgG, IgA) 2. T-cell subpopulation index levels before and after treatment (CD3^+^, CD4^+^) 2. Complication incidence (Anorexia, hyperhidrosis, poor sleep)	NR	6 months
Guo et al. 2016	RCT	92 (62/30) 3.80 ± 1.50	16.0 ± 2.0 (months)	11.0 ± 1.0 (times/year)	1.YPFG (children < 20 kg, 2.5 g, TID;≥20 kg, 5 g, TID) 2. Routine treatment	1. Pidotimod Tablets (400 mg, BID; after 14 days, 400 mg/day) 2. Routine treatment	2–3 months	1. Total effective rate 2. Duration of clinical symptoms (fever,cough,lung rales, pharynx) 3. Duration of antibiotic therapy 4. Recurrence rate within 6 months	No significant adverse events	6 months
Ma et al. 2012	RCT	110 (55/55) T:4.7 ± 3.2 C:4.6 ± 3.1	1–2 (years)	T: 7–9 C: 8–10 (times/year)	1. YPFG (children ages 1–3, 2.5 g, TID; > 3 years old, 5 g, TID) 2. Routine treatment	1. Routine treatment	4 months	1. Total effective rate	NR	1 year
Fu et al. 2012	RCT	171 (75/96) T:7.56 ± 0.89 C:7.81 ± 1.07	T:3.34 ± 0.79 C:3.26 ± 0.74 (years)	T:8.75 ± 0.93 C:8.62 ± 0.86 (times/year)	1. YPFG (children ages 2–5, 2.5 g, TID; 5-10 years old, 5 g, BID; >10 years old, 5 g, TID) 2. Routine treatment	1. Placebo (Dosage is same) 2. Routine treatment	1 month	1. T-cell subpopulation index levels before and after treatment (CD4^+^, CD8^+^, CD4^+^/CD8^+^) 2. Serum immunoglobulin levels before and after treatment (IgA, IgG, IgM)	NR	1 year
Liang et al. 2021	RCT	100 (57/43) T:6.24 ± 1.12 C:5.25 ± 2.16	T:2.97 ± 0.23 C:2.58 ± 0.15 (years)	NR	1. YPFG (5 g,TID) 2. Routine treatment	1. Routine treatment	4 weeks	1. Total effective rate 2. Recurrence rate within 1 year 3. Duration of clinical symptoms (fever, cough, red throat, lung rales)	NR	1 year
Zhang 2021	RCT: double -blind	100 (59/41) T:4.54 ± 0.79 C:4.51 ± 0.78	T:2.13 ± 0.45 C:2.11 ± 0.46 (years)	T:9.96 ± 1.44 C:9.94 ± 1.46 (times/year)	1. YPFG (Children ages 1–3, 5 g/day; 4–6 years old,7.5 g/day; TID) 2. Pidotimod oral solution (400 mg, QD or BID) 3. Routine treatment	1. Routine treatment	8 weeks	1. Total effective rate 2. T-cell subpopulation index levels before and after treatment (CD3^+^, CD4^+^, CD8^+^) 3. Serum immunoglobulin levels before and after treatment (IgA, IgG, IgM) 4. STCMS 5. Inflammatory factor levels (IL-2, IL-6, IL-8, TNF-*α*) 6. Pulmonary function index (FEV1, FVC, PEF)	NR	NR
Tian et al. 2016	RCT: single -blind	100 (51/49) T:5.51 ± 2.37 C:5.79 ± 2.77	T:1.94 ± 0.97 C:2.14 ± 0.82 (years)	NR	1. YPFG (children ages 1–3, 5 g/day; ages 4–6, 7.5 g/day; Ages 7–9, 10 g/day; ages 10–14, 12.5 g/day, TID) 2. Routine treatment	1. Pidotimod granule (0.4 g, BID; after 2 weeks,0.4 g, QD) 2. Routine treatment	3 months	1. Total effective rate 2. Serum immunoglobulin levels before and after treatment (IgA, IgG, IgM) 3. Inflammatory factor levels (TNF-α, IL-2)	No significant adverse events	NR
Lin et al. 2020	RCT	120 (59/61) T:4.67 ± 1.64 C:4.48 ± 1.53	T:2.18 ± 0.35 C:2.21 ± 0.42 (years)	T:7.84 ± 1.98 C:8.06 ± 1.86 (times/year)	1. YPFG (children ages > 6, 5 g,TID; ages 4–6, 5 g, BID; ages 1–3, 2.5 g,BID) 2.Control	1. Ribavirin tablets: 10 mg/ (kg·d),TID 2. Routine treatment	7 days	1. Total effective rate 2. Number of infections before and after therapy 3. T-cell subpopulation index levels before and after treatment (CD3^+^, CD4^+^, CD8^+^, CD4^+^/CD8^+^)	NR	NR
Yan 2019	RCT	100 (57/43) T:6.0 ± 0.6 C:6.0 ± 0.6	T:1.93 ± 0.25 C:1.89 ± 0.23 (years)	T:10.4 ± 1.3 C:10.3 ± 1.4 (times/year)	1. YPFG (1.5–2.5 g, TID) 2. Control	1. Pidotimod dispersible tablets (0.4 g/day) 2. Routine treatment	2 months	1. Total effective rate 2. Serum immunoglobulin levels before and after treatment (IgA, IgG, IgM) 3. Duration of clinical symptoms (Fever, cough, pulmonary sounds) 4. Average time to recurrence 5. Inflammatory factor levels (IL-2, IL-6, TNF-α) 6. Incidence of adverse reactions	No obvious adverse events T:2 (6%) Slight diarrhea C:2 (4%) Slight diarrhea	1 year
Zhu 2022	RCT	90 (55/35) T:4.2 ± 0.7 C:4.1 ± 0.6	T:20.9 ± 3.4 C:21.6 ± 3.3 (months)	T:6.95 ± 1.17 C:6.91 ± 1.19 (times/year)	1. YPFG (children ages 1–3, 2.5 g, TID;; >3 years, 5 g,TID) 2. Routine treatment	1. Mannatide oral solution (5 ml, TID) 2. Routine treatment	2 months	1. Total effective rate 2. STCMS 3. Serum immunoglobulin levels before and after treatment (IgA, IgG, IgM) 4. Number and duration of illnesses in the year before and after treatment	No significant adverse events	1 year
Wang 2019	RCT: double-blind	70 (39/31) T:5.41 ± 1.86 C:5.69 ± 1.74	T:3.14 ± 0.52 C:3.25± 0.71 (years)	T:8.71 ± 1.68 C:8.59 ± 1.74 (times/year)	1. YPFG Children <10 kg,2.5 g, BID; 20–30 kg, 2.5 g, TID; >30 kg, 5 g,TID 2. Routine treatment	1. Routine treatment: Ribavirin, 10–15 mg/kg, 10–15 mg/kg, Add 5% dextrose Solution (100–250 ml), ivgtt, QD	T:3 months C: 9–15 days	1. Total effective rate 2. Serum immunoglobulin levels before and after treatment (IgA, IgG, IgM) 3. T-cell subpopulation index levels before and after treatment (CD4^+^, CD8^+^, CD4^+^/CD8^+^) 4. Number of infections in 1 year	NR	1 year
Hu 2020	RCT	100 (62/38) T:6.18 ± 2.35 C:6.37 ± 2.44	T:7.11 ± 1.60 C:7.28 ± 1.75 (duration of each infection, days)	T:5.25 ± 1.79 C:5.63 ± 1.55 (times/year)	1. YPFG (children ages 1–3, 1/3 packet, TID; 4–7 years old, 1/2 packet,TID; > 7 years old,1 packet,TID.5 g/packet) 2. Routine treatmen	1. Routine treatment	1 month	1.Total effective rate 2. Pre- and post-treatment classification score, respiratory symptom score, TCM symptom score 3. Immune substance levels before and after treatment (SIgA, HBD-2, SIL-2R, TNF-α) 4. Trace element levels before and after treatment (Cu, Zn, Ca, Mg, Fe)	No significant adverse effects	NR
Xu et al. 2022	RCT: multicenter, double-blind, double-simulation	271 (165/106) T:4.5 ± 1.3 C:4.5 ± 1.3	NR	T:10.07 ± 3.94 C:9.82 ± 3.35	YPFG and sham pidotimod oral solution: 2.5 g to children aged 2–3 years, or at 5 g to children aged 4–6 years; once in the morning and once in the evening.	pidotimod and sham YPF granules:400 mg once daily, 1 h after dinner.	8 weeks	1. Total effective rate 2. Reduction value of respiratory tract infection frequency 3. Traditional Chinese medicine symptom disappearance rate 4. Adverse events 5. Pharmacoeconomic analysis	T: No drug-related adverse events C:2 (rhinorrhea, rash)	12 months

STCMS, traditional Chinese medicine evidence score; NR, no report; QD, medication once a day; QOD, medication every other day; BID, medication twice a day; TID, medication three times a day.

### Statistical analysis and results

#### The total clinical effective rate

**Figure 4:**
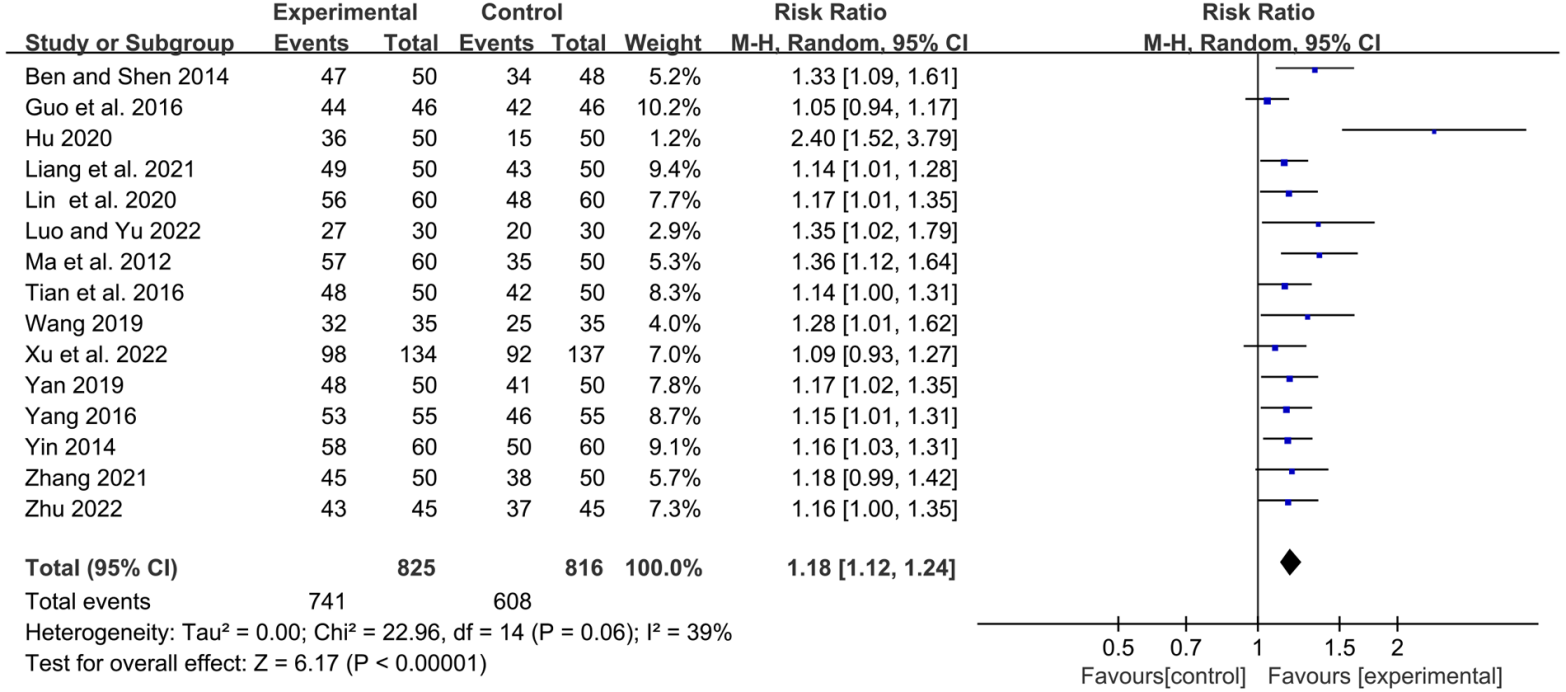
Forest plot of the total clinical response rate.

The frequency of RTI and the degree of symptom relief after treatment are the main indicators for evaluating the clinical efficacy of RRTIs in children. Moreover, the definition and calculation methods of the total clinical response rate differ due to the different definitions and diagnostic basis of diseases. Fifteen studies reported the total clinical response rate, so we included these data in the meta-analysis. Statistics showed that the total response rate in the YPFG group was significantly higher than that in the control group [RR = 1.18, 95%CI (1.12, 1.24)]. However, there may be potential heterogeneity among studies (*P* = 0.06, *I*^2 ^= 39%) (Figure 4). Sensitivity analysis found no significant change in the data (Supplementary Figure S1). We performed a subgroup analysis of the included studies according to the treatment characteristics of the experimental and control groups and the duration of treatment in the experimental group to analyze the sources of heterogeneity in the results. Courses are broadly divided into two categories: within one month and for at least two months. The experimental group could be divided into YPFG treatment and YPFG combined with immunomodulators. The control group was divided into two categories according to whether the immunomodulator was used or not. Subgroup analysis showed that the total response rate was higher in the YPFG group when the treatment was less than one month [RR = 1.22, 95%CI (1.09, 1.37), *P* = 0.0008, *I*^2^ = 67%]. When the treatment time was more than two months, the total response rate of YPFG treatment was significantly higher than that of conventional therapy [RR = 1.16, 95%CI (1.10, 1.23), *P* < 0.00001, *I*^2^ = 17%]. Subgroup analysis showed that the total response rate of YPFG adjuvant therapy was higher than that of the control group [RR = 1.17, 95%CI (1.10, 1.25), *P* < 0.00001, *I*^2 ^= 49%]. The total response rate of YPFG combined with immunomodulators was significantly better than that of the control group [RR = 1.23, 95%CI (1.12, 1.34), *P* < 0.0001, *I*^2 ^= 0%]. According to the characteristics of the control group, the subgroup analysis showed that the total response rate of the YPFG group was significantly higher than that of the control group using an immunomodulator [RR = 1.14, 95%CI (1.07, 1.21), *P* < 0.0001, *I*^2 ^= 12%]. The total response rate of the YPFG group was significantly better than that of the control group without immunomodulators [RR = 1.22, 95%CI (1.12, 1.33), *P* < 0.0001, *I*^2 ^= 56%] (Table 2).

**Table 2:** The subgroup analysis of RRTIs in children treated with adjuvant YPFG therapy.

				Heterogeneity		
	NO	RR (95%CI) or SMD (95%CI)	P within group	P heterogeneity	I^2^	P between sub-groups
**Subgroup analysis of YPFG for overall clinical efficacy.**						
Overall effect	15	1.18[1.12,1.24]	<0.00001	0.06	39%	
Control group type						
No immunomodulator	8	1.22 [1.12, 1.33]	<0.0001	0.03	56%	
With immunomodulators	7	1.14 [1.07, 1.21]	<0.0001	0.33	12%	0.23
Test group type						
YPFG	11	1.17 [1.10, 1.25]	<0.00001	0.03	49%	
YPFG combined with immunomodulators	4	1.23 [1.12, 1.34]	<0.0001	0.63	0%	0.4
Trial duration (month)						
One month	6	1.22 [1.09, 1.37]	0.0008	0.01	67%	
Two months	9	1.16 [1.10, 1.23]	< 0.00001	0.29	17%	0.47
**Subgroup analysis of YPFG for IgA.**						
Overall effect	12	1.23 [0.68, 1.78]	< 0.0001	< 0.00001	95%	
Control group type						
No immunomodulator	5	1.73 [0.74, 2.72]	0.0006	< 0.00001	96%	
With immunomodulators	7	0.88 [0.23, 1.52]	0.007	< 0.00001	94%	0.15
Test group type						
YPFG	7	1.34 [0.35, 2.34]	0.008	< 0.00001	97%	
YPFG combined with immunomodulators	5	1.04 [0.78, 1.31]	< 0.00001	0.09	51%	0.57
Trial duration (month)						
One month	4	2.11 [0.87, 3.35]	0.0009	< 0.00001	96%	
Two months	8	0.81 [0.27, 1.34]	0.003	< 0.00001	92%	0.06
**Subgroup analysis of YPFG for IgM.**						
Overall effect	11	0.85 [0.35, 1.35]	0.0009	< 0.00001	93%	
Control group type						
No immunomodulator	5	1.30 [0.70, 1.89]	< 0.0001	< 0.00001	90%	
With immunomodulators	6	0.48 [-0.22, 1.17]	0.18	< 0.00001	93%	0.08
Test group type						
YPFG	7	0.77 [0.04, 1.50]	0.04	< 0.00001	95%	
YPFG combined with immunomodulators	4	0.98 [0.36, 1.60]	0.002	< 0.0001	87%	0.67
Trial duration (month)						
One month	4	1.40 [0.69, 2.12]	0.0001	< 0.00001	91%	
Two months	7	0.53 [-0.09, 1.16]	0.09	< 0.00001	93%	0.07
**Subgroup analysis of YPFG for IgG.**						
Overall effect	12	1.06 [0.65, 1.47]	< 0.00001	< 0.00001	91%	
Control group type						
No immunomodulator	5	1.37 [0.91, 1.82]	< 0.00001	0.0001	82%	
With immunomodulators	7	0.85[0.25, 1.44]	0.005	< 0.00001	93%	0.17
Test group type						
YPFG	7	1.12 [0.42, 1.82]	0.002	< 0.00001	95%	
YPFG combined with immunomodulators	5	0.98 [0.67, 1.29]	< 0.00001	0.03	64%	0.72
Trial duration (month)						
One month	4	1.56 [1.19, 1.92]	< 0.00001	0.04	64%	
Two months	8	0.82 [0.31, 1.33]	0.002	< 0.00001	92%	0.02
**Subgroup analysis of YPFG for TNF-α.**						
Overall effect	4	-1.03[-1.55,-0.51]	0.0001	0.0004	84%	
Control group type						
No immunomodulator	2	-0.79[-1.22,-0.36]	0.0003	0.14	55%	0.40
With immunomodulators	2	-1.29[-2.37,-0.21]	0.02	0.0005	92%	
Test group type						
YPFG	2	-1.20[-2.45, 0.04]	0.06	< 0.0001	94%	0.61
YPFG combined with immunomodulators	2	-0.87[-1.16,-0.58]	< 0.00001	0.37	0%	
Trial duration (month)						
One month	1	-0.57 [-0.97, -0.17]	0.005			0.10
Two months	3	-1.19 [-1.82, -0.57]	0.0002	0.002	84%	
Abbreviations: CI, confidence interval; RR,risk ratio; SMD, Std mean difference.						

#### IgA

**Figure 5:**
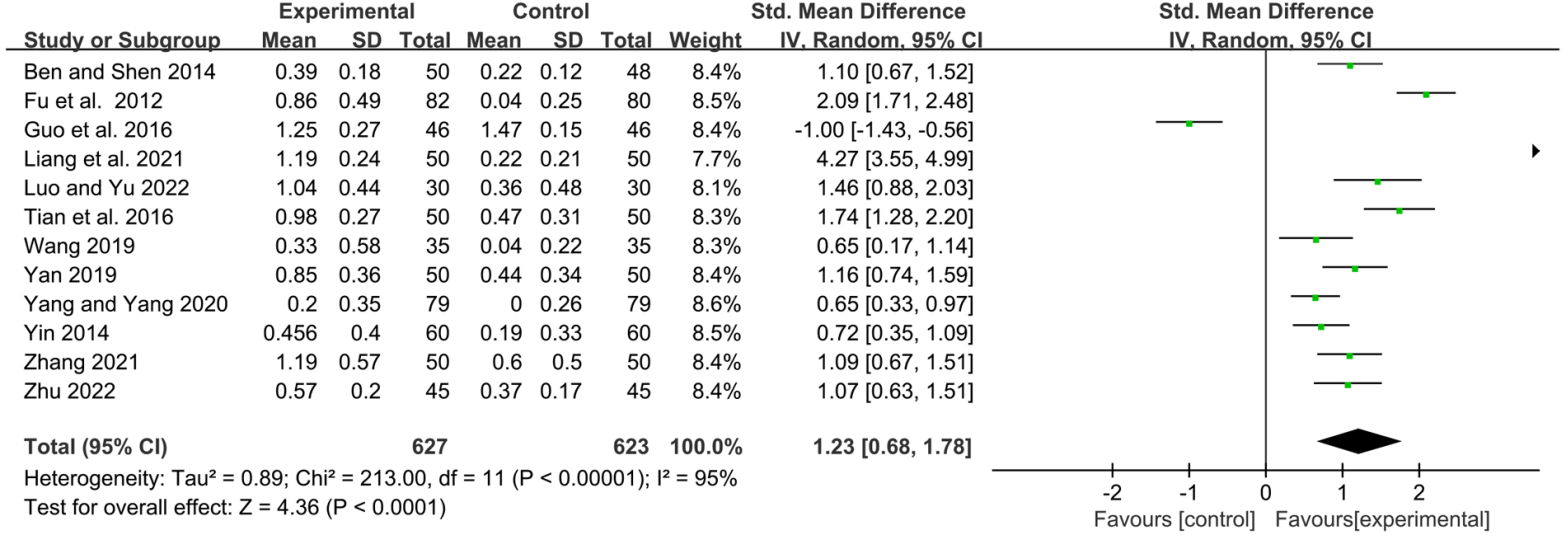
Forest plot of IgA.

Twelve studies observed serum immunoglobulin IgA levels before and after treatment. Statistics showed that YPFG treatment could significantly increase the level of serum IgA [SMD = 1.23, 95%CI (0.68, 1.78), *P* < 0.0001, *I*^2 ^= 95%] (Figure 5). Sensitivity analysis suggested that the results had good stability (Supplementary Figure S2). In terms of the duration of treatment, within 1 month of treatment, the serum IgA levels in the YPFG group increased more significantly [SMD = 2.11, 95%CI (0.87, 3.35), *P* = 0.0009, *I*^2 ^= 96%]. After more than 2 months of treatment, YPFG treatment could significantly improve IgA level [SMD = 0.81, 95%CI (0.27, 1.34), *P* = 0.003, *I*^2 ^= 92%]. From the classification of the experimental group, subgroup analysis showed that YPFG adjuvant therapy could increase the level of serum IgA compared with conventional therapy [SMD = 1.34, 95%CI (0.35, 2.34), *P* = 0.008, *I*^2 ^= 97%]. Compared with conventional therapy, YPFG combined with immunomodulators can significantly increase the level of serum IgA [SMD = 1.04, 95%CI (0.78, 1.31), *P* < 0.00001, *I*^2 ^= 51%]. Regarding the classification of the control group, the subgroup analysis showed that the serum IgA level in the YPFG group increased significantly compared with the control group using immunomodulators [SMD = 0.88, 95%CI (0.23, 1.52), *P *= 0.007, *I*^2 ^= 94%]. Compared with the control group without immunomodulators, the serum IgA level in the YPFG group was significantly increased [SMD = 1.73, 95%CI (0.74, 2.72), *P* = 0.0006, *I*^2 ^= 96%] (Table 2).

#### IgM

**Figure 6:**
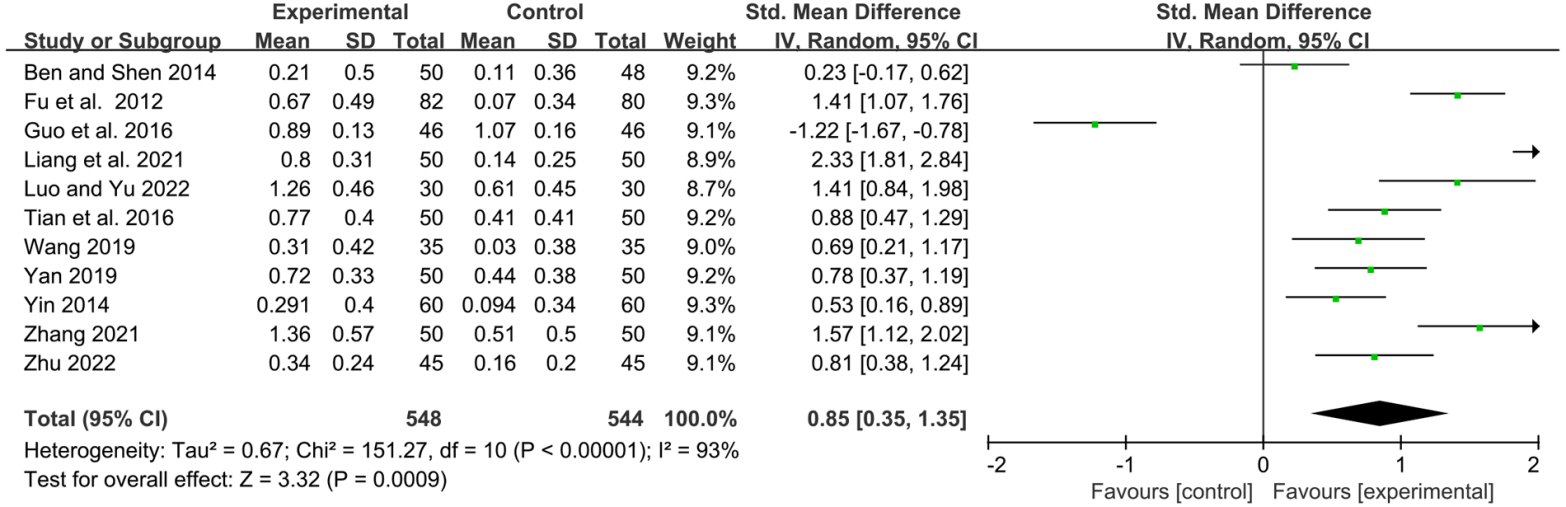
Forest plot of IgM.

Eleven studies examined serum immunoglobulin IgM levels before and after treatment. Statistics showed that YPFG treatment significantly increased IgM levels [SMD = 0.85, 95%CI (0.35, 1.35), *P* = 0.0009, *I*^2 ^= 93%] (Figure 6). Sensitivity analysis showed that the data were basically stable. However, the study by Guo et al. (22) may be a source of heterogeneity (Supplementary Figure S3). Compared with the control group receiving immunomodulatory treatment, subgroup analysis showed no significant difference between the two groups [SMD = 0.48, 95%CI (−0.22, 1.17), *P* = 0.18, *I*^2 ^= 93%]. Compared with the control group without immunomodulators, YPFG treatment significantly increased the level of IgM [SMD = 1.30, 95%CI (0.70, 1.89), *P* < 0.0001, *I*^2 ^= 90%]. According to the classification analysis of the experimental group, YPFG adjuvant treatment was better than the control group [SMD = 0.77, 95%CI (0.04, 1.50), *P* = 0.04, *I*^2 ^= 95%]. YPFG combined with immunomodulators increased the serum IgM levels compared with the control group [SMD = 0.98, 95%CI (0.36, 1.60), *P* = 0.002, *I*^2 ^= 87%]. Within one month of treatment, YPFG treatment significantly improved the level of IgM [SMD = 1.40, 95%CI (0.69, 2.12), *P* = 0.0001, *I*^2 ^= 91%]. There was no significant difference in the efficacy of the YPFG group and conventional treatment when the treatment duration was more than two months[SMD = 0.53, 95%CI (−0.09, 1.16), *P* = 0.09, *I*^2 ^= 93%] (Table 2).

#### IgG

**Figure 7:**
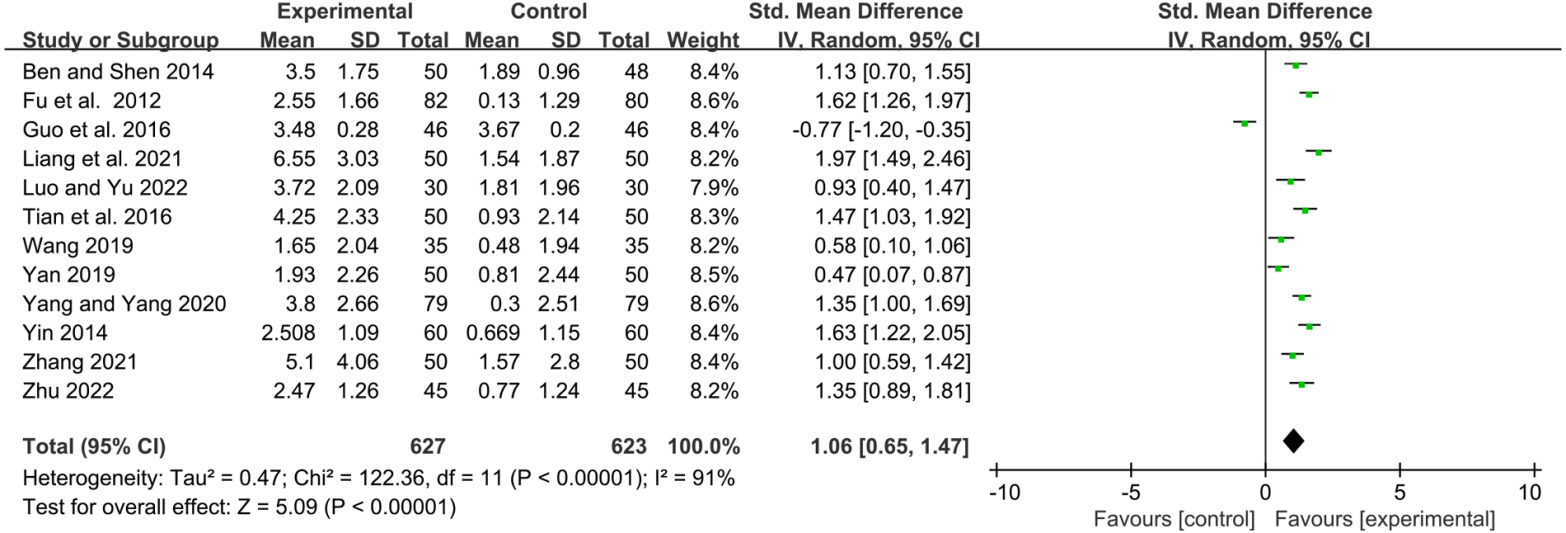
Forest plot of IgG.

Twelve studies reported serum immunoglobulin IgG levels before and after treatment. Statistical analysis showed that the YPFG group could significantly increase serum IgG levels [SMD = 1.06, 95%CI (0.65, 1.47), *P* < 0.00001, *I*^2 ^= 91%] (Figure 7). Sensitivity analysis results were more stable but suggested that the study by Guo et al. (22) may be a source of heterogeneity (Supplementary Figure S4). Subgroup analysis showed that the YPFG group increased the serum IgG levels compared that the control group with immunomodulator [SMD = 0.85, 95%CI (0.25, 1.44), *P* = 0.005, *I*^2 ^= 93%]. Compared with the control group without immunomodulator, YPFG could significantly increase the level of serum IgG [SMD = 1.37, 95%CI (0.91, 1.82), *P* < 0.00001, *I*^2 ^= 82%]. Subgroup analysis showed that compared with the control group, YPFG treatment could significantly increase the level of the serum IgG [SMD = 0.85, 95%CI (0.25, 1.44), *P* = 0.005, *I*^2 ^= 93%], YPFG combined with immunomodulators also significantly increased serum IgG level [SMD = 0.98, 95%CI (0.67, 1.29), *P* < 0.00001, *I*^2 ^= 64%]. The YPFG group could significantly increase the level of serum IgG within one month of treatment [SMD = 1.56, 95%CI (1.19, 1.92), *P* < 0.00001, *I*^2 ^= 64%]. After more than 2 months of treatment, the level of serum IgG increased significantly in the YPFG group [SMD = 0.82, 95%CI (0.31, 1.33), *P* = 0.002, *I*^2 ^= 92%] (Table 2).

#### TNF-α

**Figure 8:**
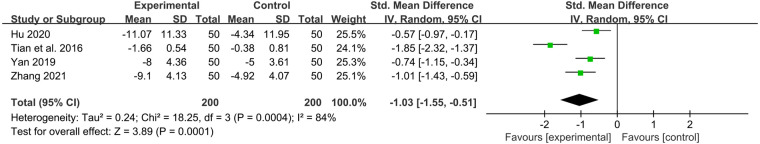
Forest plot of TNF-α.

Four studies reported the values of TNF-α before and after treatment. YPFG treatment significantly reduced TNF-α level [SMD = −1.03, 95%CI (−1.55, −0.51), *P* = 0.0001, *I*^2 ^= 84%] (Figure 8). Sensitivity analysis results were more stable, but Tian et al. (33) may be a source of heterogeneity (Supplementary Figure S5). Subgroup analysis showed that TNF-α levels decreased significantly after YPFG treatment regardless of whether the control group received an immunomodulator or not. On the other hand, YPFG combined with immunomodulators significantly reduced TNF-α levels compared with the control group. The TNF-α level in the YPFG group was significantly decreased by subgroup analysis of treatment time (Table 2).

### Adverse event

Nine studies described the observation of adverse events, three of which recorded the proportion of adverse events and specific symptoms: Luo and Yu (42) found one case each of nausea and vomiting in the experimental group (6.67%), three cases of nausea, and five cases of vomiting in the control group, for a total of eight cases (26.67%). Yan (44) found there were two cases of mild diarrhea in the experimental group (6%) and the control group (4%). Xu et al. (23) found no drug-related adverse events in the experimental group, while there were two cases (1.46%) in the control group, manifested as rhinorrhea and skin rash. None of the above adverse events severely affected the participants, so the study results were complete. No significant adverse events were found in the remaining studies.

### GRADE evidence quality assessment

We assessed the quality of the evidence for each result, as shown in Supplementary File S2. The quality of evidence for total clinical response rate and TNF-α levels were rated as low. In contrast, the quality of evidence for serum immunoglobulin IgA, IgG, and IgM levels was rated as very low, respectively.

### Publication bias

**Figure 9:**
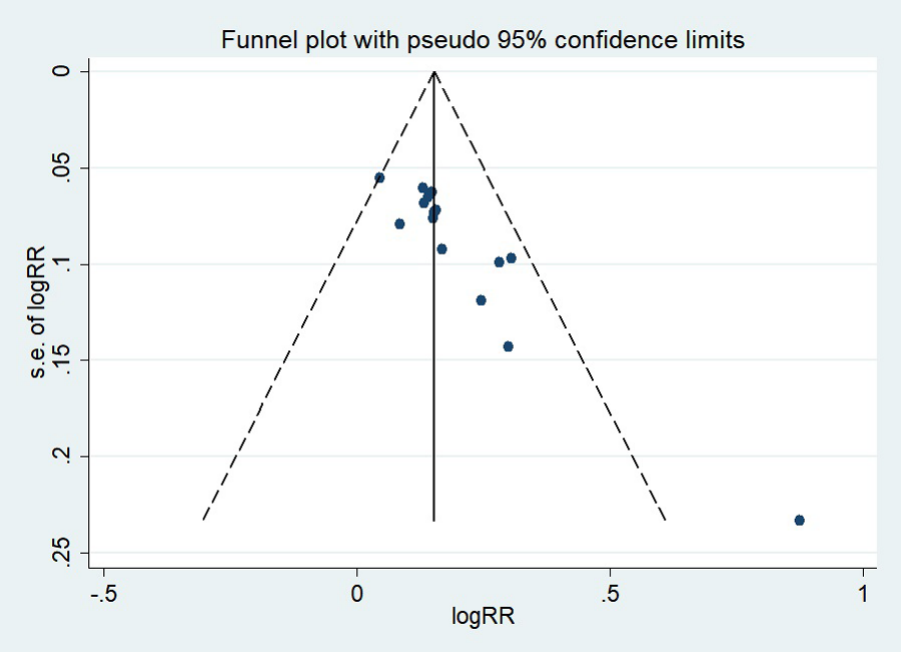
Funnel plot of the total clinical response rate.

**Figure 10:**
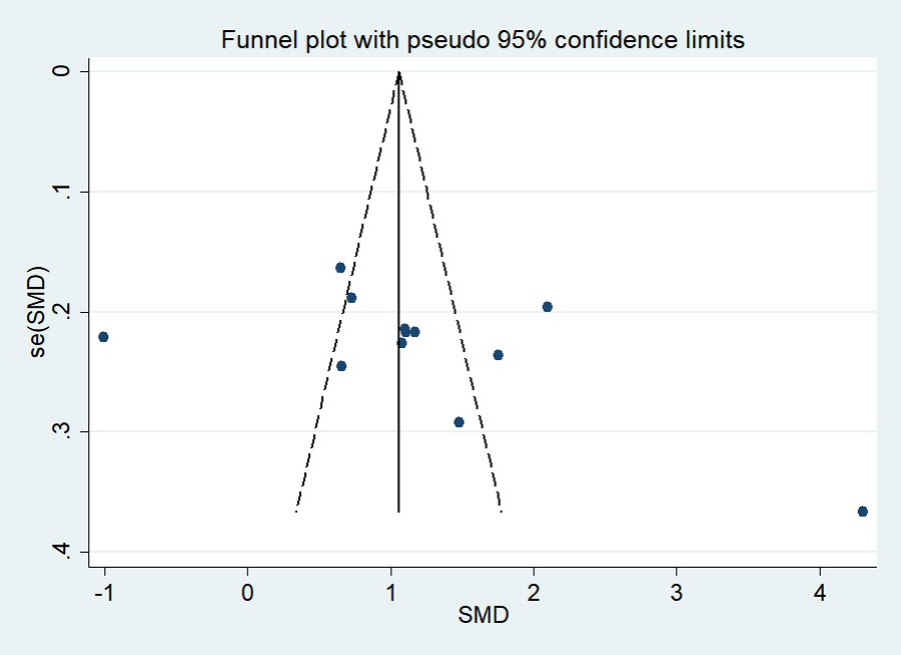
Funnel plot of IgA.

**Figure 11:**
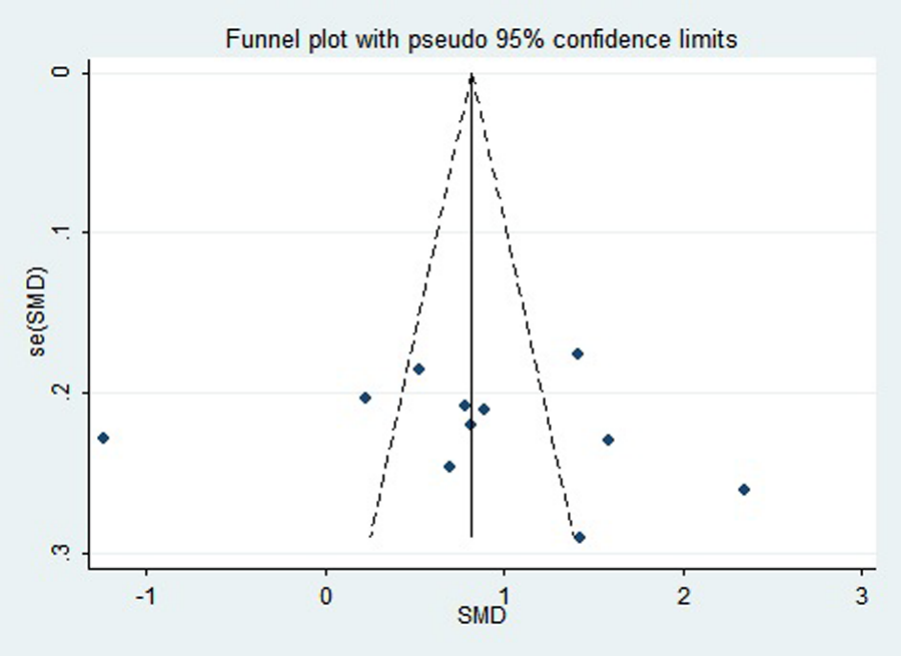
Funnel plot of IgM.

**Figure 12:**
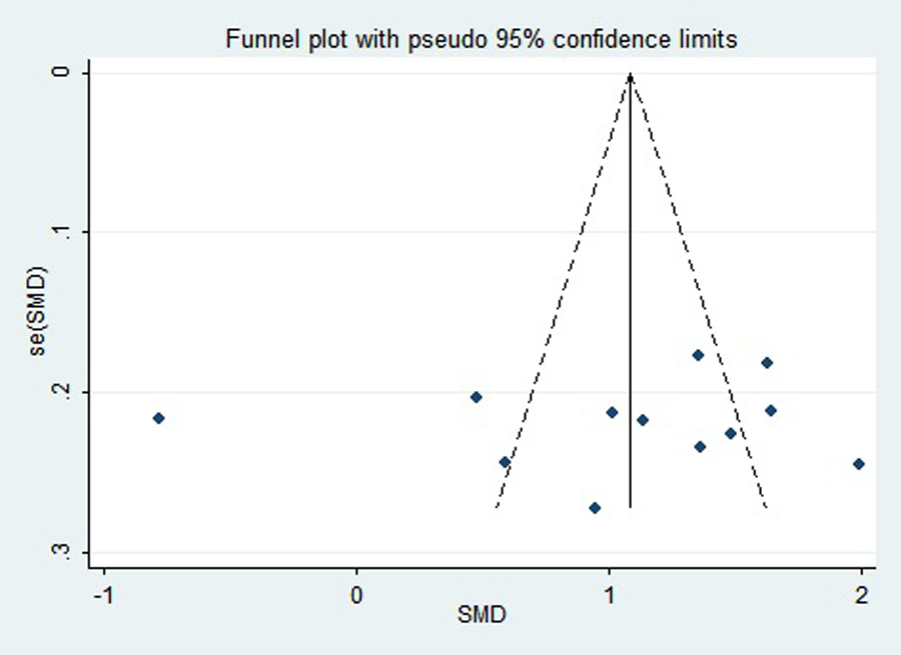
Funnel plot of IgG.

First, regarding the total clinical response rate, we found asymmetry through the visual inspection of the funnel plot (Figure 9) and using Egger's (*P* = 0.000) regression test, and found significant publication bias, thus assessing the combined effect size of the total clinical response rate using the trim and fill method, which showed a statistically significant difference between the two groups. The combined results were stable (Supplementary Figure S6). Next, we observed asymmetry based on the funnel plot of IgA (Figure 10), and no significant publication bias was found using Egger's (*P* = 0.123) regression test. Then, by visual inspection of the funnel plot of IgM, we found no significant asymmetry (Figure 11), and no significant publication bias was found using Egger's (*P* = 0.644) regression test. Finally, we also found no significant asymmetry based on visual inspection of the funnel plot of IgG (Figure 12), and no significant publication bias was found using Egger's (*P* = 0.731) regression test.

## Discussion

This study conducted a meta-analysis of current clinical trials using YPFG as adjuvant therapy for pediatric RRTIs to provide strong evidence. Studies have shown that adjuvant YPFG therapy can improve the overall clinical response rate, increase serum immunoglobulin IgA, IgM, and IgG levels, and significantly decrease TNF-α levels, thereby reducing the rate of infection recurrence and improving children's immunity. As there was significant heterogeneity between studies, a subgroup analysis was used to explore the possible sources of heterogeneity. The comparison of medication and time between the two groups showed that adjuvant YPFG treatment could significantly improve overall clinical response rate, serum IgA, IgM, and IgG levels. According to the treatment characteristics of the control group and the intervention time of the study, adjuvant YPFG therapy was more favorable in reducing tumor necrosis factors. According to the classification of the drugs used in the experimental group, when YPFG was used alone, there was no significant difference in the changes in TNF- *α* level between the YPFG group and the control group. When YPFG was combined with immunomodulators, the YPFG group could significantly reduce the TNF-α level.

Based on the results of subgroup (Table 2) and sensitivity analyses (Supplementary File S3), we believe that Hu's study and Guo et al.'s study may be the source of heterogeneity. By looking at baseline characteristics of RCTs, we found that 62 boys and 38 girls in the Hu study reported the duration of each episode of childhood respiratory infection, suggesting possible clinical heterogeneity. In the study by Guo et al., the sample size was smaller and included 62 boys and 30 girls. Immunomodulators were used in the control group and at higher doses than in other clinical trials, suggesting possible clinical heterogeneity.

Pediatric RRTIs endanger the physical and mental health of children, and are accompanied by a heavy medical and economic burden, and are an important health problem of global concern (3). Pediatric RRTIs are closely related to airway anatomy and physiology and transient immune weakness. The younger the child, the lower the location of helper T cells and the lower the level of secretory IgA and IgG, especially the IgG subclass. In addition, respiratory infection is caused by various factors, such as lack of trace elements and nutrients, repeated infection with pathogens, etc. (46).

Although deficiencies in the immune system trigger the development of RRTIs in children, the specific pathogenesis remains unclear, and as a result, no evident biological agents have been approved for clinical treatment (10).

It is worth noting that TCM has long established that children with RRTIs are characterized by a deficiency of both the lung and spleen, and the most common symptoms are fatigue, sweating, recurrent colds, and respiratory discomfort. YPFG is a prescription commonly used in treating children with deficiency of lung and spleen syndrome in traditional Chinese medicine. It has the effect of tonifying Qi, reducing perspiration, and strengthening the exterior. YPFG, as one of the preparations included in the Pharmacopoeia of the People's Republic of China, has the function of two-way immune regulation and can be widely used in the treatment of chronic diseases such as asthma and respiratory infections, including secondary immune deficiency in children (47, 48). By the way, YPFG is considered a potential immune booster in TCM (23). Ma et al. (18) found that through conventional treatment, YPFG significantly relieved clinical symptoms, prevented acute exacerbations, and improved symptom scores in COPD patients with good safety, which may be related to the anti-fatigue and anti-hypoxia functions of astragalus (49). Animal experiments show that Yupingfeng (YPF) can improve alveolar-capillary barrier damage induced by exhaustive exercise in rats by modulating the cytoskeleton (50).

Experiments have shown that YPF can improve the function of the thymus and spleen, increase the activity of B lymphocytes, T lymphocytes, and NK cells, counteract the apoptosis of lymphocytes and strengthen immunity (51). Studies have shown that YPF can reduce the expression of Bcl2L12, promote the balance of T helper cells 1/2, and regulate immune function in patients with allergic rhinitis (52). YPF has a variety of active components that inhibit the type 2 response mediated by Group2 innate lymphoid cells (ILC2s) (53), blocking influenza virus (IFV) and human respiratory syncytial virus (HRSV) entry into the airways and can relieve airway inflammation, thereby reducing lung injury and improving survival (54). Polysaccharide (YPF-PS) isolated from YPF could significantly increase the proliferation and phagocytic capacity of macrophages, increase the levels of cytokines such as nitric oxide and tumor necrosis factor, and increase the expression of CD4^+^ and CD8^+^ T cells. In addition, YPF-PS could increase the level of serum antibodies and promote the proliferation of T lymphocytes. YPF extensively regulates immunity, anti-inflammatory, and antiviral infections (55).

Notably, YPFG contains astragalus (AR, Huangqi), the main active components of which are polysaccharides, saponins, and flavonoids, which can promote the maturation of acquired immune cells, enhance antibodies and improve innate immune function (56). Studies have confirmed astragalus, combined with other herbal treatments, can reduce the risk of acute respiratory infection (ARTIS) in children (57). The mechanism of YPFG regulating immune function is related to bile acid and glycerol phospholipid metabolism, and the active components of YPFG can affect this metabolism and inhibit inflammation (58).

To our knowledge, the meta-analysis by Song et al. reported that the YPF formula could increase serum IgA, IgG, IgM, and CD3^+^ levels in children with RRTIs (59). A meta-analysis by Zhao et al. showed that YPFG could significantly improve the IgA, IgG, CD3^+^, CD4^+^ levels, and CD4^+^/CD8^+^ ratio in children, but randomized methods were not mentioned (60). A meta-analysis by Zhang et al. has demonstrated that routine treatment in combination with YPFG or conventional therapy in combination with YPFG and Pidotimod can improve the total response rate and immunoglobulin levels in children with RRTIs (61).

In our meta-analysis, for the first time, the specificity of the intervention drug was used as an essential inclusion criterion for literature screening, and only studies with perfect drug information were considered. In addition, the experimental group used YPFG, excluding YPF oral liquid or powder to reduce sources of heterogeneity of drug composition differences. Moreover, we recently conducted multicenter, double-blind design clinical studies and clinical trials using blinded randomization, which facilitated the accuracy of the pooled data compared to previously published meta-analyses. Our study summarizes a new metric. TNF-α, although only four studies reported this data. TNF-α has physiological functions, such as regulating immune responses and promoting cell growth and differentiation. TNF-α is an early inflammatory transmitter, which can impair the function of the upper respiratory tract in children, interfere with the immune regulation mechanism, and cause recurrent respiratory tract infections. It is an important indicator of inflammation. In addition, seven studies have shown that conventional adjuvant therapy with YPFG significantly reduces the frequency of recurrent respiratory infections in children. Six studies reported the benefit of adjuvant treatment with YPFG in increasing the levels of T-cell subsets in children. No severe side effects of YPFG treatment were reported in our included literature, thus careful long-term safety monitoring needs to be considered.

In conclusion, YPFG adjuvant therapy may improve overall clinical efficiency, increase serum IgG, IgM, and IgA levels, and decrease TNF-α levels. Due to the use of multiple combination drug interventions in the included clinical trials, there may be significant clinical heterogeneity and the clinical efficacy of YPFG adjuvant therapy in children with RRTIs should be viewed with caution.

## Limitations

Despite the novelty of this meta-analysis, some limitations need to be considered. The included clinical trials were all conducted in China, and only one study was published in English, so there may be regional differences in the results of the studies. The methodological quality of the study design was generally low, and only one clinical trial used a double-blind design. Therefore, well-designed, more rigorously designed clinical trials are needed to investigate the efficacy of YPFG adjuvant therapy. In addition, adverse events regarding YPFG were not reported in some studies, which may affect the objective evaluation of the efficacy of YPFG. Some studies did not report the duration of follow-up, which may affect the results of subgroup analysis.

## Conclusion

Overall, the results of the current systematic review and meta-analysis suggest that YPFG adjuvant therapy improves the overall clinical response rate, increases serum IgA, IgM, and IgG levels, and reduces TNF-α levels in pediatric RRTIs. However, due to the design flaws of some of the included studies, more multicenter, double-blind RCTs are needed to support the veracity of the results of this study.

## Data Availability

The original contributions presented in the study are included in the article/**Supplementary Material**, further inquiries can be directed to the corresponding author.
