# Supplementary material for: Adjuvant treatment with yupingfeng granules for recurrent respiratory tract infections in children: A systematic review and meta-analysis
Source: Front Pediatr. 2022 Dec 21;10:1005745. doi: 10.3389/fped.2022.1005745 (PMC9811950; doi:10.3389/fped.2022.1005745)
Supplement: Supplementary file 1 [file Datasheet1.zip › Datasheet5/Table1.docx]

Table 1. Characteristics of the included literatures.

| **Study** | **Type** | **Participant characteristics** | | | **Test** | **Control** | **Treatment  duration** | **Outcomes** | **Adverse**  **events** | **Follow-up** |
| --- | --- | --- | --- | --- | --- | --- | --- | --- | --- | --- |
|  |  | Sample size, sex(boy/girl),  Age(T/C), year | Disease  duration | Recurrence  times of infection |  |  |  |  |  |  |
| Yin 2014 | RCT | 120(63/57)  T:31.37±7.817  C:30.05±8.096  (months) | T:3.03±1.339  C:3.03±1.615  (Pre-hospital course, days) | T:5.196±1.271  C:4.966±1.160  (times/ half a year) | 1.YPFG  (children ages 1-3, 2.5g, TID; >3 years old, 5 g, TID)  2. Routine treatment | 1. Routine treatment | 1 month | 1. Total effective rate  2. STCMS  3. Signs and Symptoms Score  4. Serum immunoglobulin levels before and after treatment(IgA、IgG、IgM)  5. Number of infections within 6 months | No significant  adverse events | 6months |
| Ben and Shen 2014 | RCT | 98(52/46)  T:3.8± 2.6  C:4.5± 2.3 | NR | NR | 1.YPFG  (Children ages < 3, 2.5 g, TID;≥3 years old, 5 g, TID)  2.Control | 1. Spleen-aminopeptide  (2mg/day, QOD)  2. Routine treatment | 2 months | 1. Total effective rate  2. Serum immunoglobulin levels before and after treatment(IgA、IgG、IgM) | NR | 1 year |
| Yang 2016 | RCT | 110(60/50)  T:2.9±1.5  C:3.1±1.7 | NR | NR | 1.YPFG  (Children ages 1-3, 2.5 g, TID;≥3 years old , 5 g, TID)  2.Routine treatment | 1. Routine treatment | 1 month | 1.Total effective rate  2.Post-treatment serum immunoglobulin levels  (IgA、IgG、IgM) | No significant  adverse events | NR |
| Luo and Yu 2022 | RCT | 60(31/29)  T:3. 80±1. 55  C:3. 51 ± 1. 74 | T:18. 64 ± 2. 59  C:17. 34 ± 3. 49  (months) | T:8. 06±1. 69  C:8. 01±2. 54  (times/year) | 1. YPFG   (5g/packet,5g,TID)  2.Control | 1.Routine treatment  2.Pidotimod oral solution (7ml,take one dose in the morning and one in the evening) | 2 weeks | 1.Total effective rate  2.Serum immunoglobulin levels before and after treatment(IgA、IgG、IgM)  3.T-cell subpopulation index levels before and after treatment (CD3^+^,CD4^+^,CD8^+^)  4.Duration of clinical symptoms (Cough, tonsillar erythema, fever and wet rales)  5.Incidence of adverse reactions | T:2(6.67%):  1nausea,1vomiting  C:8(26. 67%):  3 cases of nausea, 5 vomiting | 6months |
| Yang and Yang 2020 | RCT | 158(82/76)  T:3.7±1.2  C:3.5±1.1 | T:14.5±3.3  C:14.2±3.1  (months) | NR | 1.YPFG  (Children< 10 kg,  1.5g,TID;10-20kg, 2.5 g,BID;20-30kg, 2.5 g, TID;≥30 kg children, 5 g, TID)  2.Calcium and zinc gluconates oral solution  3.Routine treatment | 1.Pidotimod granule  (20 mL,BID；after 2 weeks,20 mL, QD)  2.Calcium and zinc gluconates oral solution  (5-10mL, BID or TID)  3.Routine treatment | 2 months | 1. Serum immunoglobulin levels before and after treatment(IgG、IgA) 2. T-cell subpopulation index levels before and after treatment (CD3^+^, CD4^+^)   2.Complication incidence  (Anorexia, hyperhidrosis, poor sleep) | NR | 6months |
| Guo et al.2016 | RCT | 92(62/30)  3.80 ± 1.50 | 16.0 ± 2.0  (months) | 11.0 ± 1.0  (times/year) | 1.YPFG  (children<20kg, 2.5g, TID;≥20kg,5g,TID)  2.Routine treatment | 1.Pidotimod Tablets  (400mg,BID; after14 days,400mg/day )  2.Routine treatment | 2-3  months | 1.Total effective rate  2.Duration of clinical symptoms (fever,cough,lung rales, pharynx)  3.Duration of antibiotic therapy  4.Recurrence rate within 6 months | No significant  adverse events | 6months |
| Ma et al. 2012 | RCT | 110(55/55)  T:4.7±3.2  C:4.6±3.1 | 1-2  (years) | T:7-９  C:8-10  (times/year) | 1.YPFG  (children ages 1-3, 2.5g, TID; >3 years old, 5g, TID)  2.Routine treatment | 1.Routine treatment | 4 months | 1.Total effective rate | NR | 1 year |
| Fu et al. 2012 | RCT | 171(75/96)  T:7.56±0.89  C:7.81±1.07 | T:3.34±0.79  C:3.26±0.74  (years) | T:8.75±0.93  C:8.62±0.86  (times/year) | 1.YPFG  (children ages 2-5, 2.5g,TID; 5-10 years old, 5g, BID; >10 years old,5g,TID)  2.Routine treatment | 1.Placebo  (Dosage is same)  2.Routine treatment | 1 month | 1. T-cell subpopulation index levels before and after treatment (CD4^+^, CD8^+^, CD4^+^/CD8^+^)   2.Serum immunoglobulin levels before and after treatment(IgA、IgG、IgM) | NR | 1 year |
| Liang et al. 2021 | RCT | 100(57/43)  T:6.24±1.12  C:5.25±2.16 | T:2.97±0.23  C:2.58±0.15  (years) | NR | 1.YPFG  (5g,TID)  2.Routine treatment | 1.Routine treatment | 4 weeks | 1.Total effective rate  2.Recurrence rate within 1 year  3.Duration of clinical symptoms  (fever, cough, red throat, lung rales) | NR | 1 year |
| Zhang 2021 | RCT:  double -blind | 100(59/41)  T:4.54±0.79  C:4.51±0.78 | T:2.13± 0.45  C:2.11±0.46  (years) | T:9.96±1.44  C:9.94±1.46  (times/year) | 1.YPFG  (Children ages 1-3, 5g/day; 4-6 years old,7.5g/day;TID)  2.Pidotimod oral solution  (400mg, QD orBID)  3.Routine treatment | 1.Routine treatment | 8 weeks | 1.Total effective rate  2.T-cell subpopulation index levels before and after treatment(CD3^+^，CD4^+^，CD8^+^）  3.Serum immunoglobulin levels before and after treatment(IgA、IgG、IgM)  4.STCMS  5.Inflammatory factor levels  (IL－2 ,IL－6、IL－8、TNF－α)  6.Pulmonary function index  (FEV1、FVC、PEF) | NR | NR |
| Tian et al.. 2016 | RCT:  single -blind | 100(51/49)  T:5.51±2.37  C:5.79±2.77 | T:1.94±0.97  C:2.14±0.82  (years) | NR | 1.YPFG  (children ages 1-3,5g/day; ages4-6,7.5g/day;  ages7-9,10g/day; ages10-14, 12.5g/day, TID)  2.Routine treatment | 1.Pidotimod granule  (0.4g, BID; after 2 weeks,0.4g, QD)  2.Routine treatment | 3 months | 1.Total effective rate  2.Serum immunoglobulin levels before and after treatment(IgA、IgG、IgM)  3.Inflammatory factor levels  (TNF－α,IL-2) | No significant  adverse events | NR |
| Lin et al. 2020 | RCT | 120(59/61)  T:4.67±1.64  C:4.48±1.53 | T:2.18±0.35  C:2.21±0.42  (years) | T:7.84±1.98  C:8.06±1.86  (times/year) | 1.YPFG  (children ages>6, 5g,TID; ages4-6,5g,BID;ages1-3,  2.5 g,BID)  2.Control | 1.Ribavirin tablets:  10 mg/(kg·d),TID  2.Routine treatment | 7 days | 1.Total effective rate  2.Number of infections before and after therapy  3.T-cell subpopulation index levels before and after treatment(CD3^+^，CD4^+^，CD8^+^,CD4^+^ /CD8^+^) | NR | NR |
| Yan 2019 | RCT | 100(57/43)  T:6.0±0.6  C:6.0±0.6 | T:1.93±0.25  C:1.89±0.23  (years) | T:10.4±1.3  C:10.3±1.4  (times/year) | 1.YPFG  (1.5-2.5g, TID)  2.Control | 1.Pidotimod dispersible tablets  (0.4g/day)  2.Routine treatment | 2 months | 1. Total effective rate 2. Serum immunoglobulin levels before and after treatment(IgA、IgG、IgM) 3. Duration of clinical symptoms (Fever, cough, pulmonary sounds) 4. Average time to recurrence 5. Inflammatory factor levels（IL-2、IL-6、TNF-α） 6. Incidence of adverse reactions | No obvious  adverse events  T:2(6%)  Slight diarrhea  C:2(4%)  Slight diarrhea | 1 year |
| Zhu 2022 | RCT | 90(55/35)  T:4.2±0.7  C:4.1±0.6 | T:20.9±3.4  C:21.6±3.3  (months) | T:6.95±1.17  C:6.91±1.19  (times/year) | 1.YPFG  (children ages 1-3,2.5g, TID;;  >3 years,5 g,TID)  2.Routine treatment | 1.Mannatide oral solution  (5ml, TID)  2.Routine treatment | 2 months | 1.Total effective rate  2.STCMS  3.Serum immunoglobulin levels before and after treatment(IgA、IgG、IgM)  4.Number and duration of illnesses in the year before and after treatment | No significant  adverse events | 1 year |
| Wang 2019 | RCT:  double-blind | 70(39/31)  T:5.41±1.86  C:5.69±1.74 | T:3.14±0.52  C:3.25±  0.71  (years) | T:8.71±1.68  C:8.59±1.74  (times/year) | 1.YPFG  Children <10 kg,2.5 g,BID;20-30kg,2.5 g,TID;>30kg,5g,TID  2.Routine treatment | 1.Routine treatment:  Ribavirin,10-15mg/kg,  10~15 mg/kg，Add 5% dextrose  Solution(100-250ml),ivgtt,  QD | T:3 months  C:9-15 days | 1. Total effective rate 2. Serum immunoglobulin levels before and after treatment(IgA、IgG、IgM) 3. T-cell subpopulation index levels before and after treatment (CD4^+^,CD8^+^,CD4^+^/CD8^+^) 4. Number of infections in 1 year | NR | 1 year |
| Hu 2020 | RCT | 100(62/38)  T:6.18±2.35  C:6.37±2.44 | T:7.11±1.60  C:7.28±1.75  (duration of each infection  , days) | T:5.25±1.79  C:5.63±1.55  (times/year) | 1.YPFG  (children ages 1-3,1/3 packet, TID;4-7years old, 1/2 packet,TID; >7 years old,1 packet,TID.5g/packet)  2.Routine treatmen | 1.Routine treatment | 1 month | 1.Total effective rate  2.Pre- and post-treatment classification score, respiratory symptom score, TCM symptom score  3.Immune substance levels before and after treatment  (SIgA，HBD-2，SIL-2R，TNF-α）  4.Trace element levels before and after treatment (Cu,Zn,Ca,Mg,Fe) | No significant  adverse effects | NR |
| Xu et al. 2022 | RCT:  multicenter, double-blind,  double-simulation | 271(165/106)  T:4.5±1.3  C:4.5±1.3 | NR | T:10.07±3.94  C:9.82±3.35 | YPFG and sham pidotimod oral solution:  2.5 g to children aged 2–3 years, or at 5 g to children aged 4–6 years; once in the morning and once in the evening. | pidotimod and sham YPF granules:400 mg once daily, 1 h after dinner. | 8 weeks | 1.Total effective rate  2.Reduction value of respiratory tract infection frequency  3.Traditional Chinese medicine symptom disappearance rate  4.Adverse events  5.Pharmacoeconomic analysis | T: No drug-related adverse events  C:2(rhinorrhea, rash) | 12months |

^[[1]](#footnote-1)^

1. STCMS：Traditional Chinese Medicine Evidence Score; NR: No report; QD: Medication once a day; QOD: Medication every other day; BID: Medication twice a day; TID: Medication three times a day. [↑](#footnote-ref-1)
