# Supplementary material for: Adjuvant treatment with yupingfeng granules for recurrent respiratory tract infections in children: A systematic review and meta-analysis
Source: Front Pediatr. 2022 Dec 21;10:1005745. doi: 10.3389/fped.2022.1005745 (PMC9811950; doi:10.3389/fped.2022.1005745)
Supplement: Supplementary file 1 [file Datasheet1.zip › Datasheet5/Supplementary FileS2.GRADE profile.docx]

Supplementary File S2.GRADE profile of adjuvant treatment with YPFG for RRTIs in children.

| Quality assessment | | | | | | Summary of findings | | |
| --- | --- | --- | --- | --- | --- | --- | --- | --- |
| Outcomes | Risk of bias | Inconsistency | Indirectness | Imprecision | Publication Bias | Number of  intervention/  control | SMD (95%CI) | Quality of  evidence |
| IgA | not serious | very serious^a^ | serious^b^ | not serious | No serious limitations | 627 /623 | 1.23(0.68, 1.78) | **⊕◯◯◯**  Very low |
| IgM | not serious | very serious^c^ | serious^b^ | not serious | No serious limitations | 548 /544 | 0.85(0.35, 1.35) | **⊕◯◯◯**  Very low |
| IgG | not serious | very serious^d^ | serious^b^ | not serious | No serious limitations | 627/623 | 1.06(0.65, 1.47) | **⊕◯◯◯**  Very low |
| TNF-α | not serious | serious^e^ | serious^b^ | not serious | No serious limitations | 200 /200 | -1.03(-1.55, -0.51) | **⊕⊕◯◯**  Low |
| Total clinical efficiency | not serious | serious^f^ | serious^b^ | not serious | No serious limitations | I:698/780 (89.5%)  C:571/771 (74.1%) | Relative (95% CI)  1.18(1.12,1.24) | **⊕⊕◯◯**  Low |

CI: confidence interval; RR: risk ratio; SMD: standardised mean difference;I: intervention;C:control

a. The test for heterogeneity is significant, and the I2 is considerable, 95%

b. Studies conducted topic with different conditions.

c. The test for heterogeneity is significant, and the I2 is considerable, 93%

d. The test for heterogeneity is significant, and the I2 is considerable, 91%

e. The test for heterogeneity is significant, and the I2 is substantial, 84%

f. The test for heterogeneity is significant, and the I2 is moderate, 39%
