# Supplementary material for: Adjuvant treatment with yupingfeng granules for recurrent respiratory tract infections in children: A systematic review and meta-analysis
Source: Front Pediatr. 2022 Dec 21;10:1005745. doi: 10.3389/fped.2022.1005745 (PMC9811950; doi:10.3389/fped.2022.1005745)
Supplement: Supplementary file 1 [file Datasheet1.zip › Datasheet5/Supplementary FileS3.Sensitivity analysis.docx]

Supplementary File S3. Sensitivity analysis.

| **Remove one study** |  |  |  |
| --- | --- | --- | --- |
| **Overall effective rate** | **Risk Ratio (95%) (Random)** | **P value for overall effect** | **I^2^** |
| Ben and Shen 2014 | 1.17[1.11,1.24] | P<0.00001 | 38% |
| Guo et al. 2016 | 1.19 [1.13, 1.25] | P<0.00001 | 26% |
| Hu 2020 | 1.16 [1.11, 1.21] | P<0.00001 | 0% |
| Liang et al. 2021 | 1.19 [1.12, 1.26] | P<0.00001 | 43% |
| Lin et al. 2020 | 1.18 [1.12, 1.25] | P<0.00001 | 44% |
| Luo and Yu 2022 | 1.17 [1.11, 1.24] | P<0.00001 | 40% |
| Ma et al. 2012 | 1.17 [1.11, 1.23] | P<0.00001 | 35% |
| Tian et al. 2016 | 1.19 [1.12, 1.26] | P<0.00001 | 43% |
| Wang 2019 | 1.18 [1.11, 1.24] | P<0.00001 | 42% |
| Xu et al. 2022 | 1.19 [1.12, 1.26] | P<0.00001 | 46% |
| Yan 2019 | 1.18 [1.12, 1.25] | P<0.00001 | 44% |
| Yang 2016 | 1.18 [1.12, 1.26] | P<0.00001 | 44% |
| Yin 2014 | 1.18 [1.12, 1.26] | P<0.00001 | 44% |
| Zhang 2021 | 1.18 [1.12, 1.25] | P<0.00001 | 44% |
| Zhu 2022 | 1.18 [1.12, 1.25] | P<0.00001 | 44% |
| **IgA** | **Std.Mean difference (95%) (Random)** | **P value for overall effect** | **I^2^** |
| Ben and Shen 2014 | 1.24 [0.64, 1.85] | P < 0.0001 | 95% |
| Fu et al. 2012 | 1.15 [0.58, 1.71] | P < 0.0001 | 94% |
| Guo et al. 2016 | 1.42 [0.96, 1.87] | P<0.00001 | 92% |
| Liang et al. 2021 | 0.97 [0.51, 1.44] | P<0.0001 | 93% |
| Luo and Yu 2022 | 1.21 [0.62, 1.80] | P < 0.0001 | 95% |
| Tian et al. 2016 | 1.18 [0.59, 1.77] | P < 0.0001 | 95% |
| Wang 2019 | 1.28 [0.69, 1.88] | P < 0.0001 | 95% |
| Yan 2019 | 1.24 [0.63, 1.84] | P < 0.0001 | 95% |
| Yang and Yang 2020 | 1.28 [0.67, 1.90] | P < 0.0001 | 95% |
| Yin 2014 | 1.28 [0.67, 1.89] | P < 0.0001 | 95% |
| Zhang 2021 | 1.24 [0.64, 1.85] | P < 0.0001 | 95% |
| Zhu 2021 | 1.24 [0.64, 1.85] | P < 0.0001 | 95% |
| **IgM** | **Std.Mean difference (95%) (Random)** | **P value for overall effect** | **I^2^** |
| Ben and Shen 2014 | 0.91 [0.37, 1.46] | P = 0.0009 | 94% |
| Fu et al. 2012 | 0.79 [0.25, 1.34] | P = 0.004 | 93% |
| Guo et al. 2016 | 1.05 [0.69, 1.41] | P=0.005 | 86% |
| Liang et al. 2021 | 0.71 [0.23, 1.18] | P = 0.004 | 92% |
| Luo and Yu 2022 | 0.80 [0.26, 1.33] | P = 0.003 | 94% |
| Tian et al. 2016 | 0.85 [0.29, 1.41] | P = 0.003 | 94% |
| Wang 2019 | 0.87 [0.32, 1.42] | P = 0.002 | 94% |
| Yan 2019 | 0.86 [0.30, 1.42] | P = 0.003 | 94% |
| Yin 2014 | 0.88 [0.32, 1.44] | P = 0.002 | 94% |
| Zhang 2021 | 0.78 [0.25, 1.31] | P = 0.004 | 94% |
| Zhu 2022 | 0.86 [0.30, 1.41] | P = 0.003 | 94% |
| **IgG** | **Std.Mean difference (95%) (Random)** | **P value for overall effect** | **I^2^** |
| Ben and Shen 2014 | 1.06 [0.61, 1.50] | P < 0.00001 | 92% |
| Fu et al. 2012 | 1.01 [0.57, 1.45] | P < 0.00001 | 91% |
| Guo et al. 2016 | 1.23 [0.97, 1.50] | P < 0.00001 | 77% |
| Liang et al. 2021 | 0.98 [0.56, 1.40] | P < 0.00001 | 91% |
| Luo and Yu 2022 | 1.07 [0.63, 1.51] | P < 0.00001 | 92% |
| Tian et al. 2016 | 1.02 [0.58, 1.46] | P < 0.00001 | 92% |
| Wang 2019 | 1.10 [0.67, 1.54] | P < 0.00001 | 92% |
| Yan 2019 | 1.12 [0.68, 1.55] | P < 0.00001 | 91% |
| Yang and Yang 2020 | 1.03 [0.58, 1.49] | P < 0.00001 | 92% |
| Yin 2014 | 1.01 [0.57, 1.44] | P < 0.00001 | 91% |
| Zhang 2021 | 1.07 [0.62, 1.52] | P < 0.00001 | 92% |
| Zhu 2022 | 1.04 [0.59, 1.48] | P < 0.00001 | 92% |
| **TNF-α** | **Std. Mean difference (95%) (Random)** | **P value for overall effect** | **I^2^** |
| Hu 2020 | -1.19 [-1.82, -0.57] | P = 0.0002 | 84% |
| Tian et al. 2016 | -0.77 [-1.02, -0.52] | P < 0.00001 | 10% |
| Yan 2019 | -1.13 [-1.84, -0.43] | P = 0.002 | 88% |
| Zhang 2021 | -1.04 [-1.78, -0.30] | P = 0.006 | 89% |
