# Supplementary material for: Adjuvant treatment with yupingfeng granules for recurrent respiratory tract infections in children: A systematic review and meta-analysis
Source: Front Pediatr. 2022 Dec 21;10:1005745. doi: 10.3389/fped.2022.1005745 (PMC9811950; doi:10.3389/fped.2022.1005745)
Supplement: Supplementary file 1 [file Datasheet1.zip › Datasheet5/Table2.docx]

Table 2. The subgroup analysis of RRTIs in children treated with adjuvant YPFG therapy.

|  |  |  |  | Heterogeneity | | |
| --- | --- | --- | --- | --- | --- | --- |
|  | NO | RR (95%CI) or  SMD (95%CI) | P within group | P heterogeneity | I^2^ | P between  sub-groups |
| **Subgroup analysis of YPFG for overall clinical efficacy.** | |  |  |  |  |  |
| Overall effect | 15 | 1.18[1.12,1.24] | <0.00001 | 0.06 | 39% |  |
| Control group type |  |  |  |  |  |  |
| No immunomodulator | 8 | 1.22 [1.12, 1.33] | <0.0001 | 0.03 | 56% | 0.23 |
| With immunomodulators | 7 | 1.14 [1.07, 1.21] | <0.0001 | 0.33 | 12% |  |
| Test group type |  |  |  |  |  |  |
| YPFG | 11 | 1.17 [1.10, 1.25] | <0.00001 | 0.03 | 49% | 0.4 |
| YPFG combined with immunomodulators | 4 | 1.23 [1.12, 1.34] | <0.0001 | 0.63 | 0% |  |
| Trial duration (month) |  |  |  |  |  |  |
| One month | 6 | 1.22 [1.09, 1.37] | 0.0008 | 0.01 | 67% | 0.47 |
| Two months | 9 | 1.16 [1.10, 1.23] | < 0.00001 | 0.29 | 17% |  |
| **Subgroup analysis of YPFG for IgA.** |  |  |  |  |  |  |
| Overall effect | 12 | 1.23 [0.68, 1.78] | < 0.0001 | < 0.00001 | 95% |  |
| Control group type |  |  |  |  |  |  |
| No immunomodulator | 5 | 1.73 [0.74, 2.72] | 0.0006 | < 0.00001 | 96% | 0.15 |
| With immunomodulators | 7 | 0.88 [0.23, 1.52] | 0.007 | < 0.00001 | 94% |  |
| Test group type |  |  |  |  |  |  |
| YPFG | 7 | 1.34 [0.35, 2.34] | 0.008 | < 0.00001 | 97% | 0.57 |
| YPFG combined with immunomodulators | 5 | 1.04 [0.78, 1.31] | < 0.00001 | 0.09 | 51% |  |
| Trial duration (month) |  |  |  |  |  |  |
| One month | 4 | 2.11 [0.87, 3.35] | 0.0009 | < 0.00001 | 96% | 0.06 |
| Two months | 8 | 0.81 [0.27, 1.34] | 0.003 | < 0.00001 | 92% |  |
| **Subgroup analysis of YPFG for IgM.** |  |  |  |  |  |  |
| Overall effect | 11 | 0.85 [0.35, 1.35] | 0.0009 | < 0.00001 | 93% |  |
| Control group type |  |  |  |  |  |  |
| No immunomodulator | 5 | 1.30 [0.70, 1.89] | < 0.0001 | < 0.00001 | 90% | 0.08 |
| With immunomodulators | 6 | 0.48 [-0.22, 1.17] | 0.18 | < 0.00001 | 93% |  |
| Test group type |  |  |  |  |  |  |
| YPFG | 7 | 0.77 [0.04, 1.50] | 0.04 | < 0.00001 | 95% | 0.67 |
| YPFG combined with immunomodulators | 4 | 0.98 [0.36, 1.60] | 0.002 | < 0.0001 | 87% |  |
| Trial duration (month) |  |  |  |  |  |  |
| One month | 4 | 1.40 [0.69, 2.12] | 0.0001 | < 0.00001 | 91% | 0.07 |
| Two months | 7 | 0.53 [-0.09, 1.16] | 0.09 | < 0.00001 | 93% |  |
| **Subgroup analysis of YPFG for IgG.** |  |  |  |  |  |  |
| Overall effect | 12 | 1.06 [0.65, 1.47] | < 0.00001 | < 0.00001 | 91% |  |
| Control group type |  |  |  |  |  |  |
| No immunomodulator | 5 | 1.37 [0.91, 1.82] | < 0.00001 | 0.0001 | 82% | 0.17 |
| With immunomodulators | 7 | 0.85[0.25, 1.44] | 0.005 | < 0.00001 | 93% |  |
| Test group type |  |  |  |  |  |  |
| YPFG | 7 | 1.12 [0.42, 1.82] | 0.002 | < 0.00001 | 95% | 0.72 |
| YPFG combined with immunomodulators | 5 | 0.98 [0.67, 1.29] | < 0.00001 | 0.03 | 64% |  |
| Trial duration (month) |  |  |  |  |  |  |
| One month | 4 | 1.56 [1.19, 1.92] | < 0.00001 | 0.04 | 64% | 0.02 |
| Two months | 8 | 0.82 [0.31, 1.33] | 0.002 | < 0.00001 | 92% |  |
| **Subgroup analysis of YPFG for TNF-α.** |  |  |  |  |  |  |
| Overall effect | 4 | -1.03[-1.55,-0.51] | 0.0001 | 0.0004 | 84% |  |
| Control group type |  |  |  |  |  |  |
| No immunomodulator | 2 | -0.79[-1.22,-0.36] | 0.0003 | 0.14 | 55% | 0.40 |
| With immunomodulators | 2 | -1.29[-2.37,-0.21] | 0.02 | 0.0005 | 92% |  |
| Test group type |  |  |  |  |  |  |
| YPFG | 2 | -1.20[-2.45, 0.04] | 0.06 | < 0.0001 | 94% | 0.61 |
| YPFG combined with immunomodulators | 2 | -0.87[-1.16,-0.58] | < 0.00001 | 0.37 | 0% |  |
| Trial duration (month) |  |  |  |  |  |  |
| One month | 1 | -0.57 [-0.97, -0.17] | 0.005 |  |  | 0.10 |
| Two months | 3 | -1.19 [-1.82, -0.57] | 0.0002 | 0.002 | 84% |  |

Abbreviations: CI, confidence interval; RR,risk ratio; SMD, Std mean difference.
