# Supplementary figures and images for: Adjuvant treatment with yupingfeng granules for recurrent respiratory tract infections in children: A systematic review and meta-analysis
Source: Front Pediatr. 2022 Dec 21;10:1005745. doi: 10.3389/fped.2022.1005745 (PMC9811950; doi:10.3389/fped.2022.1005745)

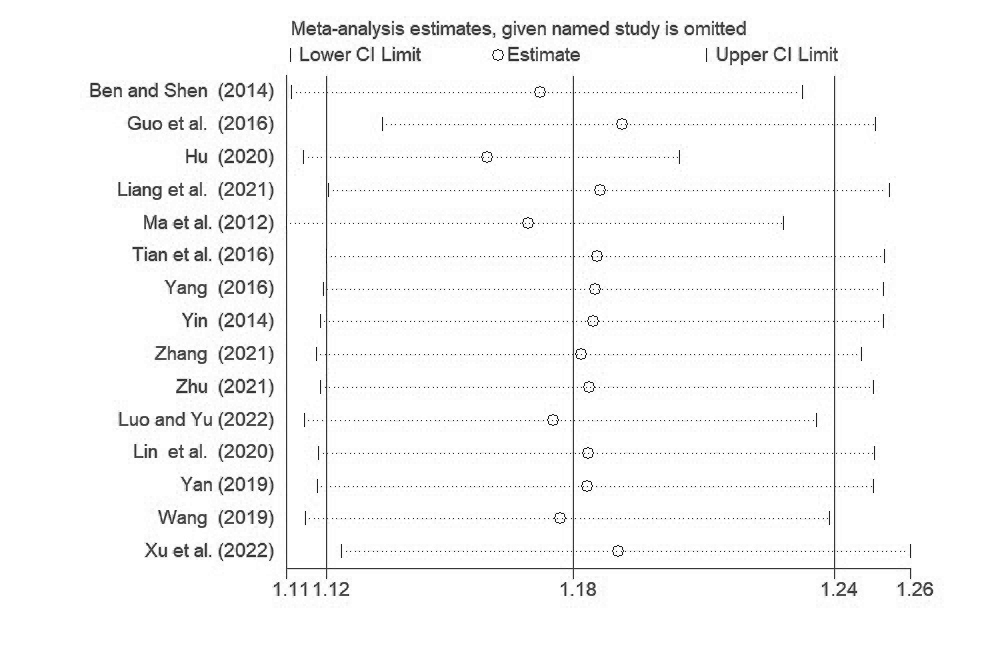

Supplement: Supplementary file 1 [file Datasheet1.zip › Datasheet5/Supplementary FigureS1.tif]

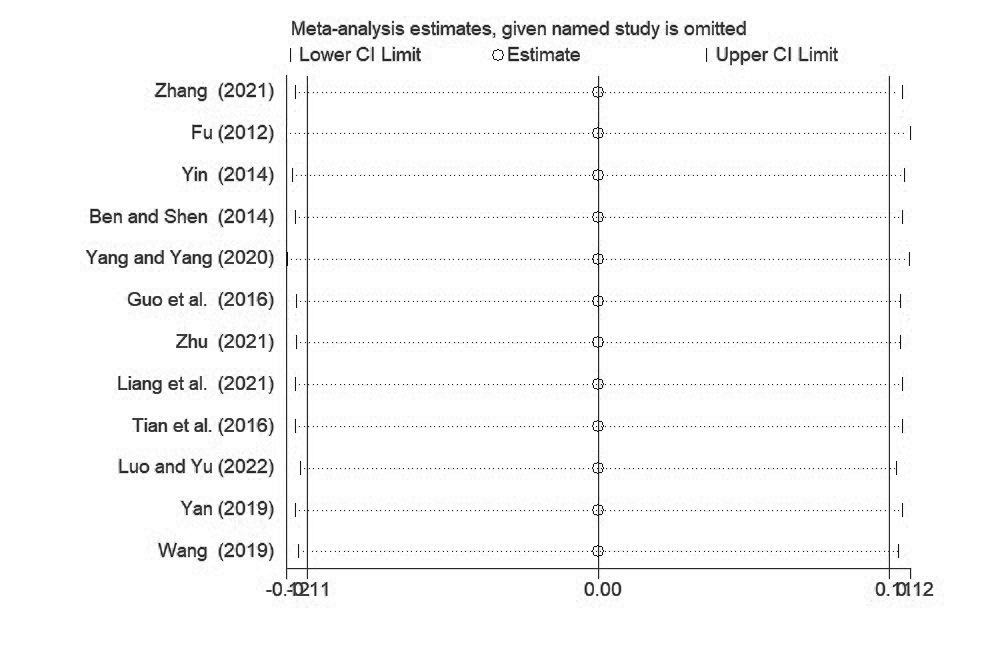

Supplement: Supplementary file 1 [file Datasheet1.zip › Datasheet5/Supplementary FigureS2.tif]

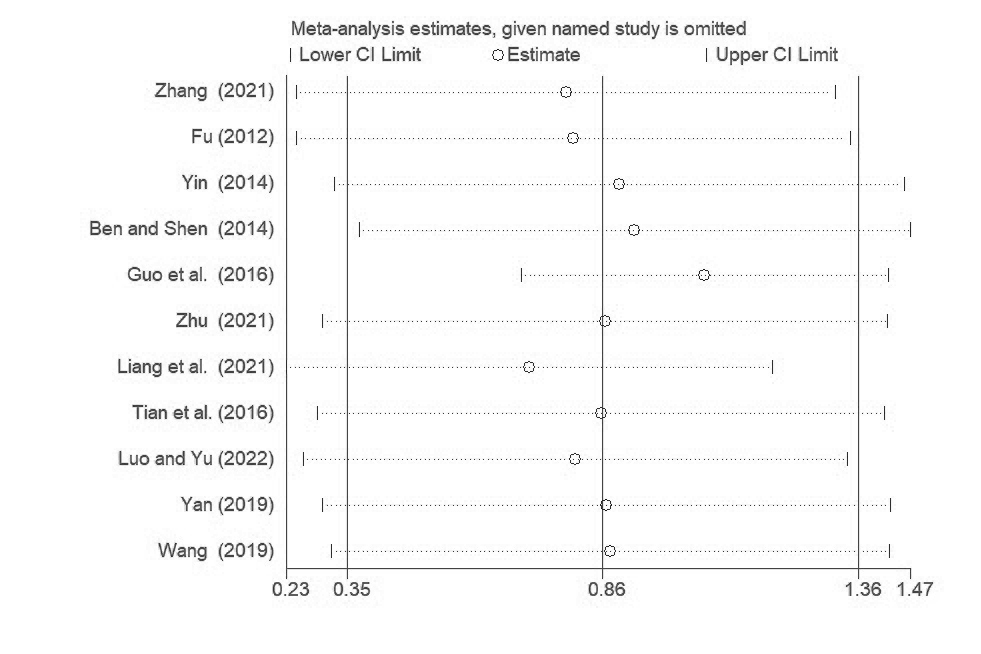

Supplement: Supplementary file 1 [file Datasheet1.zip › Datasheet5/Supplementary FigureS3.tif]

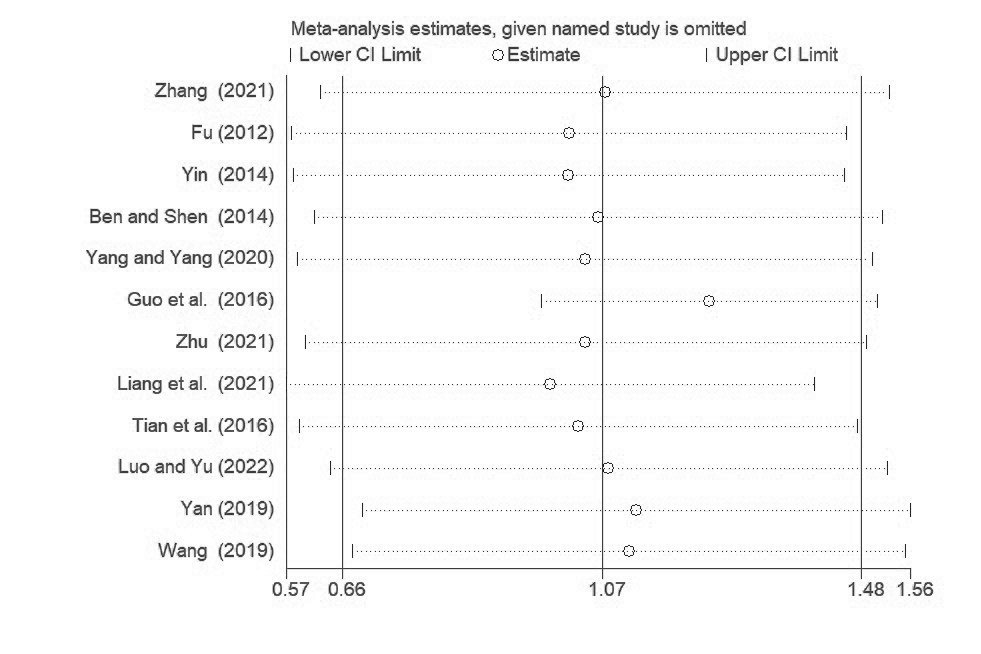

Supplement: Supplementary file 1 [file Datasheet1.zip › Datasheet5/Supplementary FigureS4.tif]

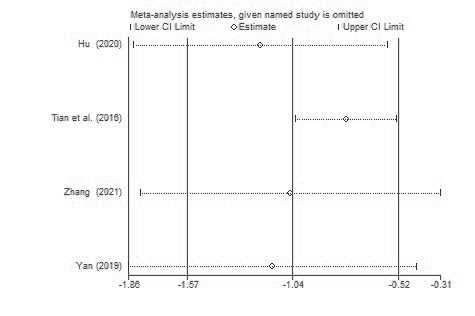

Supplement: Supplementary file 1 [file Datasheet1.zip › Datasheet5/Supplementary FigureS5.tif]

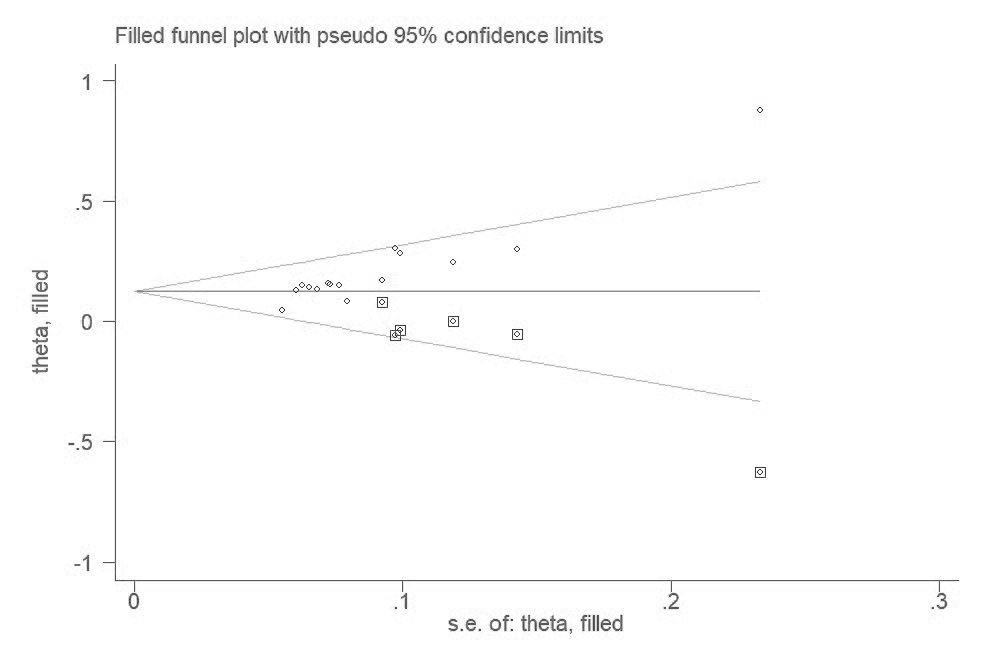

Supplement: Supplementary file 1 [file Datasheet1.zip › Datasheet5/Supplementary FigureS6.tif]
